# Patient Characterization Protocols for Psychophysiological Studies of Traumatic Brain Injury and Post-TBI Psychiatric Disorders

**DOI:** 10.3389/fneur.2013.00091

**Published:** 2013-07-22

**Authors:** Paul E. Rapp, Brenna M. Rosenberg, David O. Keyser, Dominic Nathan, Kevin M. Toruno, Christopher J. Cellucci, Alfonso M. Albano, Scott A. Wylie, Douglas Gibson, Adele M. K. Gilpin, Theodore R. Bashore

**Affiliations:** ^1^Department of Military and Emergency Medicine, Uniformed Services UniversityBethesda, MD, USA; ^2^Aquinas, LLCBerwyn, IL, USA; ^3^Physics Department, Bryn Mawr CollegeBryn Mawr, PA, USA; ^4^Neurology Department, Vanderbilt UniversityNashville, TN, USA; ^5^Combat Casualty Care Directorate, Army Medical Research and Materiel CommandFort Detrick, MD, USA; ^6^Arnold and Porter, LLPWashington, DC, USA; ^7^Department of Epidemiology and Preventive Medicine, University of MarylandCollege Park, MD, USA; ^8^Psychology Department, University of Northern ColoradoGreeley, CO, USA

**Keywords:** cognitive assessment, neuropsychiatric assessment, resilience, sociological assessment

## Abstract

Psychophysiological investigations of traumatic brain injury (TBI) are being conducted for several reasons, including the objective of learning more about the underlying physiological mechanisms of the pathological processes that can be initiated by a head injury. Additional goals include the development of objective physiologically based measures that can be used to monitor the response to treatment and to identify minimally symptomatic individuals who are at risk of delayed-onset neuropsychiatric disorders following injury. Research programs studying TBI search for relationships between psychophysiological measures, particularly ERP (event-related potential) component properties (e.g., timing, amplitude, scalp distribution), and a participant’s clinical condition. Moreover, the complex relationships between brain injury and psychiatric disorders are receiving increased research attention, and ERP technologies are making contributions to this effort. This review has two objectives supporting such research efforts. The first is to review evidence indicating that TBI is a significant risk factor for post-injury neuropsychiatric disorders. The second objective is to introduce ERP researchers who are not familiar with neuropsychiatric assessment to the instruments that are available for characterizing TBI, post-concussion syndrome, and psychiatric disorders. Specific recommendations within this very large literature are made. We have proceeded on the assumption that, as is typically the case in an ERP laboratory, the investigators are not clinically qualified and that they will not have access to participant medical records.

## Introduction

The assessment of mild TBI presents significant challenges. This is particularly true in those cases where the patient is asymptomatic or minimally symptomatic in the immediate post-injury period and subsequently presents a serious neuropsychiatric disorder. This has motivated the search for physiological variables, including alterations in the properties of ERPs (event-related potentials), which can identify individuals at risk of illness while in the premorbid state.

We wish to outline the specific aims of this contribution. It is not our present purpose to identify a comprehensive assessment procedure for traumatic brain injury (TBI). Such an assessment would include elements of the pre-injury medical history, laboratory results (biomarkers, genomics, neuroendocrine evaluation, markers of inflammation), quantitative electroencephalography, evoked potentials, event-related potentials, electromyography, eye tracking, balance assessments, a neurological examination, a psychiatric interview, and the results of imaging studies. Our purpose is far more limited. Hundreds, if not thousands, of standardized patient self-report instruments have been used in TBI studies. The diversity of instruments used has made it impossible to compare the results of different studies in a statistically meaningful way. Our object was to review the instruments that have been used and evaluate the clinical and statistical evidence that supported their use. Our recommendations, which are principally directed to psychophysiologists who are not necessarily familiar with this material, are based on this review. While this paper is primarily directed to the psychophysiological community, the recommendations may be useful in other types of TBI research such as imaging or biomarker studies. We explicitly recognize that no set of recommendations will be applicable to all studies. Investigators must make choices that will be informed by the study’s objects and clinical population. It is hoped that these recommendations may be helpful when making study-specific choices.

**Figure 1:**
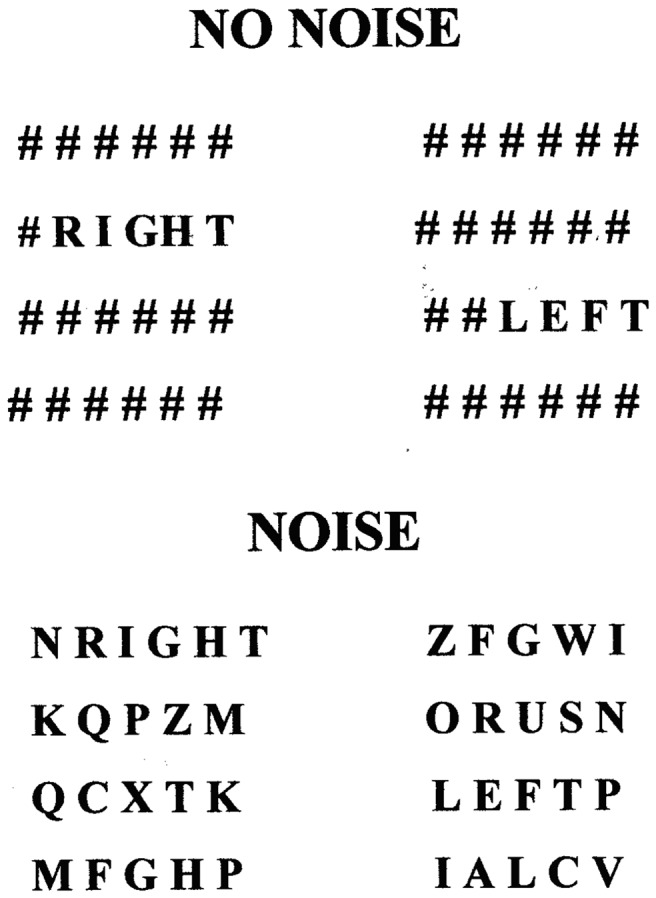
**Examples of high discriminability (NO NOISE) and low discriminability (NOISE) stimulus matrices used in the McCarthy–Donchin matrix task [modified from McCarthy and Donchin**(1)**]**.

As part of the effort to construct psychophysiological characterization of TBI, it is necessary to identify relations between experimentally induced factor effects on candidate assessment measures like ERPs and clinically observable manifestations of injury. This is particularly important in longitudinal studies where the ability of ERPs to provide indices of the responses to treatment or the progression of disease is being investigated. Variations in the levels of experimental factors (i.e., independent variables) can produce different effects on two or more dependent measures. A classic example is provided in the McCarthy and Donchin (1) matrix task in which both stimulus discriminability and stimulus-response compatibility are varied. In the matrix task visual stimuli are presented on a computer. A single trial has two components, a cue word (SAME or OPPOSITE) followed by a matrix containing the word LEFT or RIGHT. Example matrices, Noise or No-Noise, are shown in Figure 1. If the cue word is SAME and the matrix contains the word LEFT, then a left button press is the correct response. If the cue word is OPPOSITE and the word LEFT appears, a right button press is the correct response. Two factors are therefore manipulated, stimulus identification and response selection. The dependent measures were reaction time (RT) and P300 latency. McCarthy and Donchin found that variations in both stimulus discriminability and S-R compatibility influenced RT; RT was prolonged by the appearance of the target word in the noise (A–Z) matrix and by the need to make an incompatible response. In contrast, P300 latency was influenced only by variations in stimulus discriminability; it was increased when the target word appeared in a noise matrix. However, P300 latency was not altered by variations in compatibility. Thus, the factor effects on RT and P300 latency were dissociated; they were not the same. Dissociations of this type provide the rationale for using a demanding technology like ERPs. They yield information not evident in RT and allow us to fractionate the stimulus input-response output process with greater precision than is afforded by reliance exclusively on response latency.

In the ideal case, the identification of systematic relations between well-delineated clinical symptoms and precisely controlled experimental factor effects would be accomplished by a review of the patient’s clinical history in which all medical records obtained in the post-injury period including neuroradiological studies are obtained. In many cases, these records are not available to researchers. In the extreme case, clinical characterization of participants published in some ERP studies of TBI is limited to the simple statement that “participants have a documented history of TBI.” The objective of this contribution is to construct a middle path between the ideal case of access to all pertinent medical records on one hand, and the absence of any patient characterization on the other hand. The construction proceeds with two assumptions. First, it is assumed that medical records will not be available to investigators. The only data they will have is what they measure themselves. Second, the assessment will be limited to standardized clinical inventories that can be administered by ERP investigators who are not physicians or licensed psychologists. The selection of neuropsychological tests to be incorporated into a battery for TBI patients is not discussed in this paper. This issue has been addressed by Bagiella et al. (2).

A head injury is an event that may lead to a disease process or processes; it is not a disease (3). Given the lack of diagnostic precision, we propose a purely operational response by recommending that all studies of TBI/PCS [post-concussion syndrome (PCS)] have four participant groups: head injury negative/head injury positive crossed against symptom negative/symptom-positive where, if possible, symptoms are assessed on the day of the ERP study. Even this seemingly robust operationalization will introduce sources of ambiguity because a participant is symptom-positive or asymptomatic depending on the symptoms assessed and on the threshold criteria used to determine symptom presence or absence. Additionally, the symptoms presented following a brain injury are not unique to head injury patients. It is therefore essential to recognize that a Head Injury Negative-Symptom-Positive group should be incorporated into the study. In studies with sufficiently large participant populations, nature of injury (for example, blast versus non-blast injury) and time after injury can also be considered in-group partitioning.

The administration of any inventory requires time. It is impractical to administer all of the inventories and tests that come to mind. We have constructed a prioritized list for studies that reflect an interest in mild TBI that may progress to the presentation of a major psychiatric disorder or clinically significant psychiatric symptoms. Studies with a particular emphasis investigating a specific hypothesis will need to include assessment instruments that speak most directly to the hypothesis. A structured evaluation is therefore suggested. If a brief instrument for a given presentation meets diagnostic threshold, it can be followed with a more detailed examination in that area if this is a focus of the investigation. In cases where we have to make a choice between equivalent or nearly equivalent instruments, we have chosen the assessment instrument that has the longest application history and the largest validating population.

## Assessments Recommended for all ERP Studies of Traumatic Brain Injury

### Demographic information

It is essential that the demographic characteristics of the participant population be described thoroughly. Demographic information should include conventional elements: age, gender, education, ethnic/racial identification, employment status, family/household status (marriage/partnership/living alone), number of children (in home/not in home), and handedness since there are important relations between handedness and cerebral laterality which may have important implications for the effects of TBI. For participants who are present or prior serving members of the armed forces, we recommend recording duty status (active duty/separated), years in military, service branch/component, highest grade/rank attained, deployments (locations and dates), and duration since separation from service. Because it is well documented that medications can affect quantitative EEGs (4, 5), EEG topography and Loreta computations (6) and event-related potentials (7, 8), a record of current medications (name of medication, dose, and date initiated) should, therefore, be included in the patient characterization. Some medications used in the past, but not currently used may have prolonged effects on cognition, affect, and EEGs/ERPs. An effort should be made to obtain a record of past medications and the date of medication termination.

### Combat exposure

In studies with active duty military personnel and veterans, an assessment of combat exposure can inform the interpretation of other measures. Keane et al. (9) (Table 10.1) have identified 11 standardized measures of combat exposure. The most commonly used is the seven item Combat Exposure Scale (10, 11). For this reason, this instrument is recommended for use in studies where combat exposure is not a central focus of the investigation. For studies where adverse military experiences are a critical interest, the more detailed Deployment Risk and Resilience Inventory should be used (12–15) (additional information can be found at the Department of Veterans Affairs, National Center for PTSD website).

The Deployment Risk and Resilience Inventory uses 104 items to construct 14 scales, two predeployment/prewar scales, 10 deployment/Warzone scales, and two postdeployment/postwar scales. The deployment/Warzone scales are Combat Experiences, Concerns about Life and Family Disruption, Deployment Social Support, Difficulty Living and Working Environment, Exposure to the Aftermath of Battle, General Harassment, Perceived Threat, Self-Report of Nuclear/Biological/Chemical (NBC) Exposures, Sense of Preparedness, and Sexual Harassment.

### Categorization of severity at the time of injury

A categorization of severity at the time of injury can be attempted, but as noted above the uncertainties associated with long delayed assessments indicate that these classifications are only an approximation. A search for relations between changes in the characteristics of EEGs/ERPs and post-concussion symptoms determined at the time of testing (described in the next section) is more likely to be scientifically fruitful. Arlinghaus et al. (16) presented a classification of TBI based on the clinical presentation at the time of injury using either the Glasgow Coma Scale (GCS), or the duration of loss of consciousness (LOC) or the duration of post-traumatic amnesia. The VA/DoD TBI Severity Classification (17) is similar to, but not identical to, the Arlinghaus et al. classification. The two classifications differ in the LOC criterion separating moderate and severe injury and in the introduction of an additional criterion (alteration of consciousness/mental state) in the DoD classification (see Table 1).

**Table 1:** **Classification of traumatic brain injury severity**.

Criteria	Mild	Moderate	Severe
**ARLINGHAUS ET AL. (16)**			
Glasgow coma scale	13–15	9–12	≤8
Loss of consciousness	30 min or less or none	30 min to 1 week	More than 1 week
Post-traumatic amnesia	Less than 24 h	More than 24 h less than 1 week	More than 1 week
**VA/DoD**			
Glasgow coma scale	13–15	9–12	≤8
Loss of consciousness	0–30 min	30 min to 24 h	More than 24 h
Post-traumatic amnesia	Less than 24 h or none	More than 24 h less than 1 week	More than 1 week
Alteration of consciousness/mental state	A moment up to 24 h	>24 h, severity based on other criteria	>24 h, severity based on other criteria

There is a lack of consensus in the literature. Greenwald et al. (18) and Rao and Lyketsos (19) have the same classification based on the GCS, but have different criteria when classification is based on LOC (see Table 2).

**Table 2:** **Comparison of TBI classification criteria**.

Class of injury	Greenwald et al. (18)	Rao and Lyketsos (19)
Mild	Cognitively altered or loss of consciousness less than 30 min	Loss of consciousness less than 30 min
Moderate	Cognitively altered or loss of consciousness 30 min to 6 h	Loss of consciousness 1–24 h
Severe	Loss of consciousness greater than 6 h	Loss of consciousness more than 24 h

This is not an exhaustive account of TBI/concussion classification systems. Cantu (20) has summarized grading systems by Nelson et al. (21), Ommaya (22), Cantu (23, 24), Colorado Medical Society (25), Jordan et al. (26), Torg (27), Roberts (28), and Kelly and Rosenberg (29). Anderson et al. (30) report that there are at least 41 different guidelines for grading mild head injury. The Mayo Classification for Traumatic Brain Injury Severity (31) establishes criteria for the categories Symptomatic (Possible), Mild (Probable), and Moderate-Severe. The strength of the Mayo classification lies in its use of multiple indicators. It can be used when a specific single indicator is not available to investigators. In their study sample (*N* = 1501 participants) at least one single measure was not available for a large number of injury events. For example, GCS scores were not available in 74% of the injury events, loss consciousness data were not available in 70%, post-traumatic amnesia was absent in 58%, and head CT was not performed in 49% of the incidents. Using available information, however, all injury events could be classified using this procedure. This system of classification is therefore particularly well suited for retrospective studies. We recommend use of the Mayo Classification for psychophysiological research in non-clinical, civilian research environments. For research with military populations, however, it may be important to relate psychophysiological variables to pre-existing military medical histories. The VA/DoD classification is warranted in these instances. We recommend using either the Mayo or the VA/DoD classification or recording both. We again note that classifications long after the time of injury based on patient report are potentially unreliable and that these assessments should be the last elements in the battery.

### Current post-concussion symptoms

Care should be exercised in the interpretation of symptoms identified using checklists of PCS. For example, Gunstad and Suhr (32) investigated the non-specificity of PCS symptom expectation and concluded that “symptom checklists for ‘PCS’ may not be useful for diagnosis.” Two sets of diagnostic criteria for PCS are available, the ICD-10 criteria for PCS (33), and the DSM-IV criteria for post-concussional disorder (PCD) (34). The ICD-10 criteria require a LOC to meet diagnostic threshold, whereas the DSM-IV criteria require “significant cerebral concussion” as evidenced by LOC, post-traumatic amnesia, or post-traumatic onset of seizures. As McCrea (35) observes, given the restrictiveness of both sets of criteria, most mild TBI patients would be excluded from diagnosis even in the presence of significant post-traumatic symptoms. The problem of selecting which of the two criteria should be used to determine the presence or absence of post-concussion symptoms is revealed by Boake et al. (36). They compared agreement observed against agreement expected by chance between the two diagnoses using the Kappa statistic and found that it was low, a value of 0.13.

Assessment is obscured further by the fact that post-concussion symptoms are not specific to head injury. Boake et al. (37) found that among patients presenting extracranial trauma, 40% met the PCS diagnostic criterion and 7% met the PCD diagnosis criterion, whereas patients with TBI had rates of 64 and 11% (see Table 3). However, Iverson (38) found that 80% of non-head injury patients reported three or more post-concussion symptoms while Gouvier et al. (39) found “no significant differences between the brain-damaged individuals and normals on items assessing self-reported memory problems, problems becoming interested in things, frequent loss of temper, irritability fatigue, or impatience.”

**Table 3:** **Comparison of post-concussion symptoms following TBI and extracranial injury Boake et al. (37)**.

	Meet DSM-IV PCD symptom criterion	Meet ICD-10 PCS symptom criterion
Traumatic brain injury, *N* = 178	19 (11%)	114 (64%)
Extracranial trauma, *N* = 104	7 (7%)	42 (40%)

The non-specificity of PCS/PCD symptoms has caused some investigators to question the utility of the diagnosis (3). In a review of the studies cited here and additional work, Smith (40) concluded: “In summary, the so-called symptoms of post-concussional syndrome are notable in that: (1) they are present in a significant number of the normal population, and (2) they are present in very significant numbers of patients who have suffered trauma not involving concussion or brain injury. Therefore, I conclude there is inadequate evidence that these symptoms meet the definition of a ‘syndrome’.” Professor Smith entitled his letter “Post-concussional symptoms, not a syndrome.” We agree. While the validity of the diagnosis is in doubt, the characterization of symptoms remains important in the search for relationships between clinical presentation and CNS electrophysiology. Precisely articulating extant symptoms at the time of testing is important irrespective of their meeting any particular diagnostic criteria.

Our review of the literature suggests that the most viable instrument for assessing current post-concussive symptom is the Rivermead Post-Concussion Symptom Questionnaire [RPQ16, (41)], a 16 item questionnaire that can be self-administered or clinician administered. It assesses the degree of symptom severity on a scale from 0 to 4 (0 = not experienced at all; 1 = no more of a problem than before injury; 2 = a mild problem; 3 = a moderate problem; 4 = a severe problem). The RPQ16 yields an aggregate score by summing all scores of two or more. The King et al. (41) test-retest reliability study of self administration of the questionnaire resulted in a Spearman correlation coefficient of 0.90. The inter-rater (inter-clinical) scoring gave a Spearman correlation of 0.87. Their study also indicated that some symptoms (namely, headaches, dizziness, noise sensitivity, forgetfulness, and poor concentration) were experienced consistently. In contrast, other symptoms were more difficult to identify and had variable expressions. These symptoms included feeling frustrated, feeling depressed, taking longer to think, and restlessness. The authors reasoned that the reliability of the aggregate score indicates that “individual symptoms may substitute for each other over time, but leave the general level of subjective experience unchanged.”

In addition to the test-retest reliability of the summed RPQ16, Eyres et al. (42) assessed its internal and external construct validity. They estimated its internal construct validity using the Rasch model (43) and its external construct validity by comparing the summed RPQ16 score with the score on the Rivermead Head Injury Follow-Up Questionnaire (44). Their results indicated that the original 16 item summed RPQ16 score did not meet Rasch internal construct validity criteria, meaning that summing individual responses into a single aggregate score could not be justified. Their analysis did reveal, however, that if three (headache, dizziness, and nausea) of the 16 elements were summed separately, then the two resulting scales did meet internal construct validity, the RPQ3 and the RPQ13. Importantly, using the Spearman rank correlation, they estimated the external construct validity of the RPQ13 to be 0.82, with individual item correlations between 0.52 and 0.71, and of the RPQ3 to be 0.62 with individual item correlations between 0.40 and 0.60. The overall test-retest reliability of the RPQ13 was 0.89 with individual item correlations between 0.59 and 0.69. The overall test-retest reliability of the RPQ3 was 0.72 with individual item correlations between 0.59 and 0.69. This pattern of results supported their recommendation that the RPQ16 be used as two distinct scales, the RPQ3 and the RPQ13. It is interesting to note the division into two independent scales resulting from statistical analysis conducted by Eyres et al. is consistent with Ryan and Warden’s classification of symptom clusters based on clinical observation (45). Ryan and Warden identified two symptom clusters, “early” symptoms that are present immediately after injury and “late” symptoms that appear days and weeks after injury. Early symptoms include drowsiness, headaches, dizziness, and nausea. The late symptoms in the Ryan–Warden classification include irritability, concentration difficulties, memory problems, headaches, fatigue, dizziness, visual disturbances, noise sensitivity, judgment problems, depression, and anxiety.

An alternative subscale structure (somatic, cognitive, emotional) using the 16 elements of the Rivermead Post-Concussion symptom scale has been proposed by Smith-Seemiller et al. (46) (see Table 4). The utility of this set of subscores was indicated by their results which showed that chronic pain patients and mild TBI patients were indistinguishable when characterized using the single summed RPQ16; however, significant between-group differences were observed when the subscales were compared. Mild TBI patient had higher scores on the cognitive subscale than did patients with chronic pain (at *p* = 0.0005), indicating greater cognitive symptoms in the mild TBI group; whereas patients with chronic pain had higher scores on the affective subscale than did patients with mild TBI (at *p* = 0.05), indicating greater affective symptoms in the chronic pain group. In contrast, the scores on the somatic subscale for the two groups were indistinguishable. It should be stressed that, as Smith-Seemiller et al. noted, these between-group differences were found in two patient populations whose subscale scores overlapped considerably. These scores cannot, therefore, accurately classify individual chronic pain and mild TBI patients, a serious deficiency when the goal is diagnostic specificity that guides therapeutic decisions for individual patients. Potter et al. (47) performed a structural equation modeling analysis of the Smith-Seemiller three-factor results. They concluded, as did Eyres et al. that PCS is not a unitary single factor syndrome, but their analysis did support the identification of separate cognitive, emotional, and somatic subscales.

**Table 4:** **Rivermead subscales**.

Subscale	Content
RPQ3	Headaches, feelings of dizziness, nausea, and/or vomiting
RPQ13	Noise sensitivity, sleep disturbance, fatigue, irritability, depressed affect, feeling of frustration, forgetfulness, poor concentration, taking longer to think, blurred vision, light sensitivity, double vision, restlessness
RPQ (cognitive)	Forgetfulness, poor concentration, taking longer to think
RPQ (emotional)	Irritability, depressed affect, feeling of frustration, restlessness
RPQ (somatic)	Fatigue, headache, dizziness, nausea, and/or vomiting, noise sensitivity, sleep disturbance, blurred vision, double vision, sensitivity to light

Based on the results of Eyres et al. (42), Potter et al. (47) and more recently Ettenhofer and Barry (48), we conclude that PCS is not a unitary syndrome. This being the case, we recommend administering the Rivermead Post-concussion Questionnaire and reporting both the RP3/RP13 identified by Eyres et al. (42) and the RPQ (Cognitive), RPQ (Emotional), and RPQ (Somatic) identified by Smith-Seemiller et al. (46). The components assigned to each factor are identified in Table 4.

### Current post-concussion severity classification

It is helpful to characterize a clinical condition in broad categories of minimal, mild, moderate, and severe. The previously described analysis of the Rivermead Post-Concussion Questionnaire indicates that PCS is not a unitary disorder. This suggests that when a severity classification is made by summing 16 individual scores, it must be interpreted with care. It would, at best, be a broad indication of clinical status. Explicitly recognizing this, Potter et al. (47) computed the cumulative frequencies of summed RPQ16 scores from their clinical sample (168 head injury patients where post-traumatic amnesia was less than 24 h, assessed 6 months after sustaining a closed skull head injury). They considered taking 75, 90, 95 limits as cut-off bands to produce the following classification.

Minimal (<75% of sample) RPQ ∈ 0–12
Mild (75–90% of sample) RPQ ∈ 13–24
Moderate (90–95% of sample) RPQ ∈ 25–32
Severe (95% of sample) RPQ > 33

A limited qualitative understanding of classification based on these cut scores can be obtained by noting that the RPQ16 score for a non-clinical sample of adults in the general population is 5.8 (49). Potter et al. state that “these bands are provisional and await further research to examine their sensitivity and specificity against general clinical populations, as well as their correspondence to quality of life and general functioning.”

### Assessment of general health at the time of the ERP study

Event-related potentials are a sensitive but non-specific indication of CNS function. They can be altered by a wide variety of medical conditions. The interpretation of ERPs should, therefore, incorporate at least a cursory assessment of the participant’s state of health at the time of recording. Health assessments fall into two general categories, disease-specific assessments and generic assessments of health. Consideration here is limited to generic assessments. Of the generic instruments now available, the SF36 (Short Form Health Survey) is the most commonly used and systematically validated (50, 51) and is recommended for ERP studies of TBI. A qualifying observation should be made. The use of the SF36, or indeed any outcome measure, in a randomized clinical trial raises additional issues (52). The selection of instruments used in randomized clinical trials should follow the COSMIN standards (53). The SF36, a generic measure of perceived health (54–56), yields an eight scale profile of functional health and well-being: physical functioning, role-physical (problems with work or daily activities as a result of physical health), bodily pain, general health, vitality, social functioning, role-emotional (problems with work or daily activities as a result of emotional problems), and mental health. Estimates of internal consistency (α coefficients) range from 0.62 to 0.94, with majority of scores equaling or exceeding 0.80. Test-retest coefficients ranged from 0.43 to 0.90 for a 6-month interval and from 0.60 to 0.81 for a 2-week interval.

### Assessment of psychiatric symptoms at time of the ERP study

The symptom checklist-90-revised (57, 58) is a psychiatric instrument which is not specific to a single disorder. The 90 item instrument is self-administered and consists of questions of the form “How much were you disturbed by ….?” The participant responds on a scale of 0 = Not at All to 4 = Extremely. The Checklist provides scores on nine dimensions and three global indices (see Table 5).

**Table 5:** **Symptom checklist-90-R subscales and global indices**.

SCL-90-R subscales	Somatization, obsessive-compulsive, interpersonal-sensitivity, depression, anxiety, hostility, phobic anxiety, paranoid ideation, psychoticism
SCL-90-R global indices	Global severity index, positive symptom total, positive symptom distress index

An extensive literature reporting on the instrument’s reliability and validity is summarized in Derogatis (59). Reliability studies resulted in Cronbach α scores for the subscales and global indices between 0.77 and 0.90. The 1 week test-retest correlations ranged from *r* = 0.78 to 0.90, and the 10 week test-retest correlations ranged from 0.68 to 0.80. Validity was established by comparisons with the Minnesota Multiphasic Personality Inventory, the Beck Depression Inventory, the Beck Anxiety Inventory, the Montgomery–Asberg Depression Rating Scale, and the Maudsley Obessional Compulsive inventory. Separate norms are available for adolescents as young as 13 years. A sixth grade reading competency is required.

### Estimation of premorbid intelligence

It has been argued that an estimate of premorbid intellectual functioning is critical to the interpretation of any post-injury assessment (60, 61). This is a matter of particular interest for ERP researchers since there is a prior literature suggesting that there are ERP correlates with intelligence [for example (62–64)]. As summarized by Franzen et al. (65) [see also (66)] there are, broadly speaking, five approaches to estimating premorbid intelligence: (1) historical data, (2) “hold-don’t hold” estimates, (3) “best performance” estimates, (4) demographic estimation, and (5) combined methods (demographic and “hold-don’t hold” or “best performance”). Franzen et al. noted that, “Although none of the methods reviewed in this paper are optimal in all situations, any one of them is probably preferable to none at all.”

#### Historical data

Historical data include information about educational and employment history obtained in clinical interviews with the patient and family members can be used to provide an approximate estimate of premorbid intelligence. Kareken and Williams (67) conducted experiments comparing clinical judgment against a quantitative procedure for estimating an IQ. Clinicians were given demographic information about hypothetical patients and asked to estimate the corresponding IQs. The same information was used in an actuarial equation to compute an IQ estimate. The clinician’s estimates were close to the computed estimates. Nonetheless, on reviewing systematic biases that lead to inaccurate clinician estimates of intellectual function, Kareken (68) recommended using quantitative methods. This is especially appropriate for ERP researchers since the clinicians participating in the Kareken and Williams study were highly experienced neuropsychologists.

#### Hold-don’t hold methods

“Hold-don’t hold” methods estimate premorbid functioning by measuring variables that are believed to be spared by the injury or disease, that is a “hold” variable is a component of intellectual functioning that is assumed to retain its premorbid value. The validity of the procedure therefore turns on the validity of this assumption. Word reading tests are a specific implementation a of “hold” estimation procedure. They estimate premorbid intelligence by determining the participant’s ability to pronounce words from a standardized reading list. The test is constructed on the assumption that reading is highly correlated with intelligence and that reading ability is resilient against disease and injury (a “hold” variable). It is further assumed that the reading of irregular words is more robust against CNS insult than the reading of regular words (69). Evidence supporting this assumption is summarized presently. Ciplotti and Warrington (70) have noted that the method can be inaccurate if the patient had a specific learning disability prior to injury or if the injury damaged areas specifically important to the process tested, for example left temporal lobe injury. Several variants have been introduced. The National Adult Reading Test [NART, (71)] is appropriate for British participants. The North American Adult Reading Test [NAART, (72)] and the American National Adult Reading Test [AMNART, (73)] were designed for use with North American participants.

#### Best performance methods

As typically implemented in a best performance estimation, several tests are administered post-injury and the highest score is used as the estimate of pre-injury ability (61). Scores on other tests are assumed to reflect post-injury deficits if they are 1.5 SD below the highest score. This procedure assumes that there is a single performance level that characterizes an individual’s competence across many areas and that the highest score on a given test reflects this overall level (the existence of a general ability factor). This assumption has, however, been challenged (74). Their results indicate that the “best performance” method can result in an overestimate of premorbid functioning. In reviewing the Mortensen et al. results, Lezak et al. (61) noted that the Mortensen results were in most instances based on the single highest score obtained by healthy control subjects in the WAIS-A (Wechsler Adult Intelligence Scale) battery of tests. Since the overall score is a weighted average of individual test scores, the highest single score will be expected to give an overestimate of the overall score. Lezak et al. recommended using a cluster of highest scores, though the criterion for selecting that score cluster is not specified.

#### Demographic methods

The demographic method can be viewed as a quantitative version of the historical method in which a regression equation uses demographic variables to compute the premorbid IQ (75, 76). The best known example is the Barona equation (76) that predicts the WAIS-R IQ using age, sex, race, education, occupation, urban/rural residence, and geographical region. However, there is controversy over the accuracy of the estimate it provides. For example, whereas, Eppinger et al. (77), using the 1984 Barona et al. equation found that the equation overestimates the IQ of normal individuals, Ryan and Prifitera (78) found that the equation under-estimated IQ when IQ was greater than 110, but it was generally reliable in the 90–109 range. A revised formula (79) has improved accuracy for populations composed primarily of Caucasians and African Americans but also shows regression to the mean [(80, 81), where Grove’s response to Veiel and Koopman, Grove (82), and the counter-response, Veiel and Koopman (83), should also be noted].

#### Combined methods

Combined methods use both demographic and current test scores to estimate premorbid IQ. Strictly speaking, the most commonly used implementation of AMNART in IQ estimation is a combined method since it includes one demographic variable (years of education) in its regression equation (73). A more systematic approach was published by Crawford et al. (84, 85) who used NART with five demographic variables (age, sex, race, education, occupation). They found that the combined method outperformed NART alone in predicting IQ. Vanderploeg and Schinka (86) used WAIS-R subtests as measures of current ability. No single subtest was specified as the “hold” measure. Rather, 33 regression formulas were constructed using the 11 WAIS-R subtests to estimate Full Scale, Verbal and Performance IQs. Because Vanderploeg and Schinka anticipated using the procedure to estimate premorbid IQ in a TBI population, they did not make a recommendation as to which of the 33 equations should be used with a specific patient. Rather, they indicated that the choice of WAIS-R subtest should be based on the patient’s injury. For example, they suggested that the WAIS-R picture completion subtest be used following left hemisphere damage, while the comprehension, information, and vocabulary subtests be considered following right hemisphere damage. In the Oklahoma Premorbid Intelligence estimation (OPIE) procedure, another combined method, Scott et al. (87) used four demographic variables (age, race, education, and occupation). Four predictions of Full Scale IQ were computed for each participant. Three were produced using the WAIS-R Vocabulary score only, the Picture Completion score only, and both the Vocabulary and Picture Completion scores along with the demographic variables. The fourth prediction used the highest WAIS-R. The OPIE procedure was tested with several clinical populations (dementia, TBI, cerebral vascular accident, neoplasm, epilepsy, and chronic pain). Estimates of premorbid IQ did not show systematic under- or over-estimation in any of these populations.

In a comparison study, Axelrod et al. (88, 89) computed predicted FSIQ (Full Scale Intelligence Quotient) scores obtained using five methods from a population of 104 neurological patients. The five methods used were:

1. The Barona equation that uses demographic data only (76).
2. BEST-3 (90) that uses demographic information and the best age-scaled score of the WAIS-R Information, Vocabulary, and Picture Completion subtests with the corresponding regression equation of Vanderploeg and Schinka (86).
3. OPIE (91), that uses raw scores from the WAIS-R Vocabulary and Picture Completion subtests and demographic information as did Vanderploeg and Schinka (86).
4. OPIE-2 (91) a variant of the OPIE method that uses the better of the two scores on Vocabulary or Picture Completion subtests if one is specifically impaired along with demographic information.
5. OPIE-R ((92), as described in (88)) provides a quantitatively based criterion for implementing OPIE-2. The Vocabulary score and demographic information are used if the age-scaled Vocabulary score is more than four points greater than the Picture Completion score, and if the picture Completion score is four points greater than the Vocabulary score, it is used with demographic information. If the point spread between the two scores is less than four, both scores and demographic information are used in the Krull et al. (91) equation.

Results obtained with the five methods were compared to the actual FSIQ. Axelrod et al. concluded that BEST-3, OPIE, OPIE-2, and OPIE-R are “equally effective approaches for premorbid estimation.”

Riley and Simmonds (93) studied 26 patients who sustained a severe TBI. They were given the NART within 12 months of injury and again at least 12 months later. If the NART score is acceptable as a “hold” variable in this population, it should be approximately constant with time. Eleven participants (4% of sample) showed an improvement of more than five IQ points. Three participants showed an improvement of 20 points. This argues against accepting NART as a “hold” variable in the severe TBI population.

Further evidence against a “hold-don’t hold” procedure in a TBI population was published by Hoofien et al. (94). They began their investigation by noting that most of the studies used to validate predictions of premorbid intelligence are studies of concurrent validity in which a predictor based on demographic variables and/or current performance is used to estimate current performance in the WAIS-R. They also note that the studies are usually performed with a healthy control population. Hoofien et al. provide a direct test of predictive validity in which current performance and demographic information was used to estimate a premorbid intelligence score in a TBI population. The premorbid measure was the Israeli military’s Primary Psychometric Rating which is administered at age 18. This score is highly correlated with WAIS and was used to compute a premorbid IQ. The population (*N* = 54) had sustained a TBI subsequent to this initial testing. Hoofien et al. used two methods to estimate premorbid IQ. The first method, denoted BEST-10, used the best score of 10 of the 11 WAIS subtests and demographic variables with the regression equations published by Vanderploeg and Schinka (86) (10 scales are used because the Hebrew version of WAIS does not include a Vocabulary subtest.). The second method, denoted BEST-2, used the best score of either the Information subtest or the Picture Completion subtest and the same Vanderploeg and Schinka regression equations. BEST-2 therefore differs from BEST-10 in implementing an *a priori* identification of two “hold” variables. Both procedures used the same demographic variables: age, gender, premorbid occupation, and premorbid education. Following Vanderploeg and Schinka, the premorbid education and premorbid occupation scores were combined to form a single socio-economic status score. The correlations between the estimates of premorbid IQs were 0.583 for BEST-2 and 0.622 for BEST-10. The difference was not statistically significant. The BEST-10 under-estimated IQ by 2.07 points (not significant) and the BEST-2 under-estimated IQ by 5.39 points (statistically significant). In the case of BEST-2, 26% of the participants had an estimated IQ more than 1 SD from the premorbid score. While with BEST-10, 6% of the patients had an estimated score more than 1 SD from the premorbid score. Hoofien et al. conclude that for this population, a best performance procedure is better than a “hold-don’t hold” method.

In contrast, positive evidence for using a “hold-don’t hold” procedure to estimate premorbid intelligence in a TBI population has been published by Green et al. (95). In the Green et al. study, 24 participants who had experienced a severe TBI were assessed 2 and 5 months post-injury. Three tests were used to measure premorbid intelligence, the Wechsler Adult Reading Test [WTAR, (96)], the Vocabulary subtest of WAIS-III, and the Matrix Reasoning subtest of WAIS-III. As in the case of AMNART, the WTAR (Wide Range Achievement Test) uses words that have an atypical grapheme to phoneme translation. Three subtest of current ability from the WAIS-III were also administered: symbol digit modalities, similarities, and block design. The scores obtained with tests of current ability improved in a manner consistent with recovery from the TBI. Performance on WTAR was stable and the WTAR-derived estimate of IQ was similar to the estimate obtained with the 1997 Crawford and Allan demographic equation (460). Green et al. concluded “Thus, converging evidence – high stability during recovery from TBI and similar IQ estimates to those of a demographic equation suggests – that the WTAR is a valid measure of premorbid IQ for TBI. Where word pronunciation tests are indicated (i.e., in patients for whom English is spoken and read fluently), these results endorse the use of WTAR for patients with TBI.”

This review of the literature supports the conclusion that the observation of Franzen et al. (65) that no method for estimating premorbid intelligence is optimal in all situations is particularly true in the case of TBI populations. The situation is further complicated by the evolution of intelligence testing. WAIS-III was released in 1997, and WAIS-IV was released in 2008. The scores obtained on these tests are highly correlated with each other and with WAIS-R. This indicates that previous studies of the comparative value of different methods for estimating premorbid intelligence are still valid, but it is necessary to use recomputed regression equations if WAIS-III or WAIS-IV scores are to be used.

For ERP researchers premorbid intelligence estimates based on WAIS-IV and WRAT-4 raise practical difficulties. Administration of these tests requires considerable time. Administration of the complete WAIS battery requires 75 min (range 60–90 min). In a research study where premorbid intelligence is not a critical measure, this may be an unacceptable participant burden. The WTAR requires 10 min. However, access to the WAIS battery and to WTAR requires clinical licensure, and most ERP researchers are not clinically qualified. Though it is normalized for WAIS-R, AMNART with the Grober and Sliwinski formula continues to be used (97). For ERP studies where assessment of injury-induced cognitive decline is not a primary focus, and in the absence of collaborators with appropriate licensure, we recommend using AMNART with the Grober and Sliwinski formula (73) and both Barona formulas (76, 79). Caution must be exercised in the interpretation of the results particularly when the three methods give divergent values. If a licensed collaborator is available, the choice between WAIS and WTAR should be based on an overall assessment of participant burden, with WTAR being the choice that minimizes that burden.

## Additional Assessments Recommended for Studies Investigating Traumatic Brain Injury and Specific Neuropsychiatric Disorders

The relationships between TBI and neuropsychiatric disorders are receiving increased attention. In those studies where these relationships are a primary focus, it is recommended that the assessment provided by the Symptom Checklist-90-R be confirmed by additional instruments. This concurs with the recommendation of Homaifar et al. (98), who writing specifically about depression following TBI, recommended that multiple means of assessment should be used when diagnosing neuropsychiatric disorders following TBI. Here we consider eight disorders where a substantial body of evidence indicates that a TBI is a significant risk factor for their presentation: depressive illness, post-traumatic stress disorder (PTSD), anxiety, psychotic disorders, sleep disorders, suicidal ideation, alcohol abuse, and substance abuse.

The recommendations presented here follow the diagnostic classifications of the DSM-IV. At present, these diagnostic groups define the assessment criteria that must be satisfied in studies of post-TBI psychopathology. It should be noted, however, that a significant revision of assessment practices may soon be required. The classical discrete disease conceptualization of psychopathology that was modeled on physical medicine is being challenged. As summarized by Smith and Oltmanns (99), “the syndromal approach may need to be jettisoned due to lack of validity.” Among others, Smith and Oltmanns have argued for an emerging consensus in which psychopathology is described along continuous, homogeneous dimensions of functioning instead of in discrete categories. This type of dimensional approach has been taken, for example, by Widiger et al. (100) who described a four-dimensional continuum for describing personality disorders and by Brown and Barlow (101) who published a dimensional model for describing anxiety and mood disorders. Therefore while the current classifications are observed here, investigators who are looking for changes in the properties of ERPs that may be associated with different types of psychopathology should recognize that the diagnostic structure on which their investigations are based is being challenged and may be discarded. Indeed, if it is found that different alterations in the morphology and timing of ERP components are associated with the different functional dimensions now being proposed (100, 101) and not with DSM-IV categories, then ERP evidence may be important in facilitating this transition.

Further cautionary observations should be made. Association, even temporally sequenced association, does not establish causation. The results summarized here indicating that TBI can lead to psychiatric disorders require further development and should be considered suggestive, certainly important, but not definitive. The identification of causal associations between TBI and psychiatric disorders is complicated by case histories that include multiple injury events, which is particularly true in military populations, and in some instances by the delayed onset of psychiatric symptoms. This time delay can be highly variable from patient to patient. Delayed-onset presentations following TBI include depression (102–106, 463), PTSD (106–110), PCS (111–113), and psychosis (114–116).

### Assessment of depression

In addition to the general considerations concerning psychiatric diagnosis outlined above, problems are encountered when assessing post-injury depression that are specific to depressive illness. Differences in the etiology of post-injury depression can be a significant complication. Depression following TBI can be a psychological response to deficits (117) or a neurologically derived consequence of failures in CNS networks (118). This complex multifactor etiology has resulted in large variation in the reported incidence of depression following TBI. Some of the reasons for which have been summarized by Kim et al. (119). Part of the variability is due to differences in the patient populations being examined. Some studies consider only mild TBI while some include patients with moderate and severe injuries. All too often the criteria for sub-typing the severity of a TBI between mild, moderate and severe are not reported explicitly. Moreover, the time interval between injury and assessment can also be a critical factor and goes unreported. It is also important to recognize that acute, transient depression can be an appropriate situational reaction to the injury. However, if the depression persists for several months following the injury then the concern is that it reflects a symptomatic expression of underlying neuropathophysiology. Between-study variability in reported incidence is also due to differences in diagnostic criteria used to diagnose depression. For example, Seel and Kreutzer (120) found a diagnosis of depression in 38% of their sample with the Beck Depression Inventory and a rate of 30% in the same sample with the Neurobehavioral Functioning Inventory Depression Scale. The concerns raised by Kim et al. (119) were echoed by Iverson (121) who argued that PCS is often misdiagnosed as depression.

In Table 6 we have listed the incidence of depressive illness following TBI as reported over the past 25 years. Arguably the most interesting aspect of the numbers in the table is their diversity. The patient inclusion criteria of the studies listed in the table differed from study to study. For example, the highest incidences were reported by Bombardier et al. (122) who excluded uncomplicated mild TBI (GCS score from 13 to 15 with no radiological abnormalities) and by deGuise et al. (123), whose patients all presented with severe TBI. Hibbard (124) who found a depression incidence of 61% did not report injury severity. The post-TBI depression population is very heterogeneous clinically. This point in emphasized by Moldover et al. (125). These authors reviewed the multiple etiological pathways that can result in post-injury depression and emphasized the need for similarly diverse clinical responses. An especially instructive example of the etiological heterogeneity of depression in TBI patients was given by Wilk et al. (126). They compared rates observed in four groups (concussions with/without LOC crossed against blast-induced versus non-blast injury). The following rates of major depression were observed: concussion with LOC following blast (21.2%), concussion with LOC following non-blast injury (15.8%), concussion without LOC following blast-induced injury (10.2%), and concussion without LOC following non-blast injury (16.0%).

**Table 6:** **Depressive illness following traumatic brain injury**.

Source	Reported incidence of depression in a post-TBI population
Brooks et al. (372)	51% *N* = 42
Varney et al. (373)	77% *N* = 120
Schoenhuber and Gentilini (374)	39% *N* = 35
Alexander (375)	25% *N* = 36
Ettlin et al. (376)	42% *N* = 26
Jorge et al. (103)	42% *N* = 66
Fann et al. (377)	26% *N* = 50
Parker and Rosenblum (378)	36% *N* = 33
Hibbard (124)	61% *N* = 100
Sliwinski et al. (146)	25% *N* = 100
Salazar et al. (379)	34% *N* = 120
Kreutzer et al. (380)	42% *N* = 722
Silver et al. (237)	11% *N* = 361
Koponen et al. (266)	26.7% *N* = 60
Jorge and Robinson (381)	51.6% *N* = 91
Seel and Kreutzer (120)	38% (Beck Depression Index), 30% NFI Depression Scale, *N* = 172
Seel et al. (382)	27% *N* = 666
Rapoport et al. (383)	15.3% *N* = 170
Ashman et al. (384)	18.6% *N* = 188
Dikmen et al. (385)	31% at 1 month, 17% 3 to 5 years, *N* = 283
O’Donnell et al. (386)	10% *N* = 363
Rapoport et al. (387)	15.3% *N* = 74
Fann et al. (132)	22.5% *N* = 135
Rowland et al. (152)	25% *N* = 51
deGuise et al. (123)	52% *N* = 46
Homaifar et al. (98)	26.7%, 30 years post-injury, *N* = 52
Bombardier et al. (122)	53.1% *N* = 559
Wilk et al. (126)	15% *N* = 3952

An alternative assessment of the relationship between TBI and depression can be obtained from a lifetime prevalence study. Holsinger et al. (105) found that the lifetime prevalence of major depression among men who had suffered a head injury in World War II was 18.5% as compared to a rate of 13.4% for a matched comparison group. However, this is probably an underestimate. Using recently developed epidemiological methods, Kruijshaar et al. (127) controlled for the influence of recall bias when responding to survey questions. They obtained an estimate of a lifetime prevalence of major depression in the general population of 20% in men and 30% in women. Though the absolute values obtained by Holsinger et al. may be underestimates, the important observation in the present context is obtained by comparing the results from the two populations.

In summary, although the reported incidence of depressive illness after suffering a head injury varies across a reasonably wide range, the overall pattern points clearly to an increase in the probability of a depressive illness emerging following such an event. Therefore, assessment of depression in the TBI-positive population is of critical importance. Based on an assessment of diagnostic sensitivity and specificity (128), Robinson and Jorge (129) concluded that “the standard DSM-IV-TR criteria are the most logical criteria to use for the diagnosis of major depression in the TBI population.” Several standardized self-report inventories for depression that are consistent with DSM-IV criteria are available. We reviewed the Patient Health Questionnaire 9, the Neurobehavioral Functioning Inventory, and the Beck Depression Inventory.

The Patient Health Questionnaire [PHQ, (130)] screens for several common mental disorders and was originally designed for use in primary care. Included within the PHQ is a nine item depression subscale, the PHQ-9, based directly on the diagnostic criteria for major depressive disorder in the DSM-IV. The nine items are presented as a series of questions. For example, “over the past 2 weeks have you been bothered by any of the following problems: little or no interest or pleasure in doing things?” Items are scored by the assignments 0 = not at all, 1 = several days, 2 = more than half the days, 3 = nearly every day. A global score is computed by summing all individual items scores. In a general medical practice population, the PHQ-9 was found to have a sensitivity of 88% and a specificity of 88% for major depression when a global score of 10 was used as the cut-off score (131). For this population, Kroenke et al. recommended cut-off scores of 5, 10, 15, and 20 for mild, moderate, moderately severe and severe depression.

Fann et al. (132) assessed the validity of the PHQ-9 for diagnosis of major depressive disorder following TBI using the Structured Clinical Interview for Diagnosis [SCID, (133)] as the gold standard for diagnostic comparison. The study population was limited to patients with a GCS score less than or equal to 12 (i.e., moderate to severe injury) or radiological evidence of acute brain abnormality. Fann et al. found that the PHQ-9 had a diagnostic specificity of 93% and a sensitivity of 89%, a concurrence with the SCID that was anticipated since the nine items in the PHQ-9 cover the “A Criterion” symptoms of depression in the DSM-IV.

An important additional problem encountered in the assessment of depression in a post-TBI population was addressed by Cook et al. (134) using the PHQ-9. Namely, many of the symptoms of TBI and PCS (fatigue, poor concentration, disturbed sleep) are also symptoms of depression. These transdiagnostic symptoms could result in an over-estimate of depression following TBI. Stated operationally, should symptoms common to both TBI and major depressive disorder be dropped from a depression screening instrument or included with a correction factor when used in a post-TBI population? This question has been addressed by Cook et al. using a Differential Item Functioning analysis (135, 136) from Item Response Theory (137). They compared responses to PHQ-9 items obtained from a primary care patient population (*N* = 3000) and from patients presenting complicated mild to severe TBI (*N* = 365) and found that no PHQ-9 item demonstrated significant Differential Item Functioning attributable to TBI. Moreover, a sensitivity analysis did not detect an inflation of PHQ-9 scores due to the cumulative effects of negligible Differential Item Functioning. Cook et al. therefore recommended using all items of the PHQ-9 when assessing depression following TBI.

The Neurobehavioral Functioning Inventory [NFI, (138)] was developed for patients with TBI. Like the PHQ, it is not specific to depression. It assesses six different sets of symptoms: depression, somatic complaints, memory/attention, communication difficulties, aggression, and motor dysfunction. Administration requires approximately 30 min. The depression scale is based on the DSM-IV criteria for depression. Seel and Kreutzer (120) compared the depression scale of the NFI against the Beck Depression Inventory [BDI (139, 140)] and the Minnesota Multiphasic Personality Inventory [MMPI-2 (141, 142)] in participants who had suffered TBIs with mean duration of unconsciousness of 4.0 ± 11.5 days and mean number of days of post-traumatic amnesia of 12 ± 26.7 days, one third of whom experienced amnesia for more than 7 days. Thus, most of the TBIs sustained by participants in this study would be classified as moderate to severe. Kreutzer et al. observed a high degree of correlation in the diagnosis of depression between the NFI and both the BDI (*r* = 0.765) and the MMPI-2 (*r* = 0.752).

Unlike the NFI and the PHQ, the BDI (139, 140) was developed specifically to assess depression. Administration requires 5–10 min. The maximum possible aggregate score is 63. When validated with psychiatric populations, the BDI was found to have high test-retest reliability [*r* = 0.96 (143)] and high internal consistency [α = 0.92, (144)]. Moreover, responses on items in the BDI have been found to correlate with those in the SCID at *r* = 0.83 and in the Hamilton Psychiatric Rating Scale for Depression at *r* = 0.71 (145, 461). Importantly, the utility of the BDI in assessing depression following TBI has been studied by several investigators. Sliwinski et al. (146) compared the diagnostic efficacy of the 1987 version of the BDI (147) with the SCID (133) and the Institute of Rehabilitation Research Symptom Checklist [TIRR, (148)], the latter of which covers symptoms related to cognition, somatic complaints, communication problems, and behavioral problems in addition to those related to depression. They found a statistically significant but small correlation (*r* = 0.30) between a SCID diagnosis of depression and the total BDI score, whereas in contrast they found a higher and significantly larger (*r* = 0.67) correlation between the total BDI score and non-depressive symptoms on the TIRR. These differences, as Sliwinski et al. reasoned, suggest that transdiagnostic somatic symptoms common to both depression and TBI influence the BDI diagnosis. A Differential Item Functioning analysis was not performed. However, with specificities at 80 and 90%, the respective sensitivities of the BDI were 36 and 20% for this population. This overall pattern of effects led Sliwinski et al. to conclude that “In fact, current findings call into question the validity of BDI as a tool for detecting clinical depression after TBI.”

However, this conclusion was not supported by Green et al. (149) in a study of TBI patients discharged from an in-patient unit who completed the BDI as part of a 24 month follow-up assessment. These investigators were specifically interested in determining the degree to which the presence of somatic symptoms in TBI that are common to depression contribute to inflating the diagnosis of depression among TBI patients when Beck diagnostic criteria are used. A Principal Component Analysis identified three-factors that accounted for most of the variance. Those related, in order of variance explained, to negative cognition and affect, negative attitudes toward self, and somatic disturbances. More patients were classified as depressed using the cognitive/affective score only than using the total BDI score. This finding argues against the conclusion that somatic disturbance items in the Beck inventory lead to an overestimate of depression in a TBI population. Cronbach’s alpha was 0.92 indicating excellent internal consistency (150). Green et al. concluded, “This study provides preliminary evidence suggesting that the BDI (Beck Depression Inventory) may be an effective screening tool for self-report depression in TBI.”

In addition to Green et al. two other studies have implemented a factor analysis of the BDI in a TBI population. Christensen et al. (151) used the original BDI (147) and found a five-factor structure. Rowland et al. (152) used the BDI-II during the immediate post-injury period. In addition to using the current version of the BDI, this study included a mix of mild/moderate (49%) and severe injury (51%) patients. Rowland et al. identified a three-factor model that was not identical to the Green et al. factorization described above. The Rowland et al. factors are Negative Self Image (20% of the variance) symptoms of Depression (18% of the variance) and Vegetative Symptoms of Depression (12% of the variance, see Table 7). The factor structure found in the TBI population was not the same as the two-factor structure, Cognitive and Somatic-Affective, found in psychiatric populations (140). They conclude that “it seems reasonable to conclude that these items (which includes a separate factor characterizing Vegetative Symptoms of Depression) are measuring something unique to the TBI sample.”

**Table 7:** **Depression subscales in a TBI population based on the Beck depression inventory-II (152)**.

BDI item	BDI item number
**NEGATIVE SELF-EVALUATION**	
Punishment feeling	6
Guilty feelings	5
Self-criticalness	8
Loss of pleasure	4
Self-dislike	7
Loss of interest	12
Past failure	3
**SYMPTOMS OF DEPRESSION**	
Loss of energy	15
Concentration difficulty	19
Sadness	1
Irritability	17
Worthlessness	14
Crying	10
Indecisiveness	13
Tiredness or fatigue	20
Pessimism	2
Suicidal thoughts	9
**VEGETATIVE SYMPTOMS**	
Changes in appetite	18
Loss of interest in sex	21
Agitation	11
Changes in sleep pattern	16

Of fundamental interest to investigations of the influence of TBI on ERP measures of cognitive processing is the relationship between the severity of the injury and the severity of the concomitant depressive symptoms. Various classifications of the severity of depressive illness based on the aggregate score obtained with different instruments have been proposed. Beck et al. (153) have partitioned depressive disorder into the following levels of severity: none or minimal depression (aggregate score less than 10), mild to moderate depression (aggregate score 10–18), moderate to severe depression (aggregate score 19–29), and severe depression (aggregate score 30–63). The appropriateness of applying these cut-off criteria to a post-TBI population was examined systemically by Homaifar et al. (98). Using signal detection theory they constructed the receiver operating characteristic (ROC) for depression following TBI with the BDI-II as the discriminating metric and the SCID as the diagnostic standard. Optimal diagnostic efficiency (87% sensitivity and 79% specificity) was obtained with an aggregate Beck score of at least 19 following mild TBI and at least 35 following moderate or severe TBI.

On the basis of the patterns of results reviewed here, our recommendation is that depression following TBI be assessed using the BDI with the Homaifar et al. (98) cutoffs of 19 following mild TBI and 35 following moderate to severe TBI. Subscales based on the Rowland et al. factor analysis (Table 7) can be included in the report in order to explore relationships between different depression factors and post-injury alterations of ERPs.

### Assessment of post-traumatic stress disorder

According to the DSM-IV, PTSD can occur in an individual who “has been exposed to a traumatic event in which both of the following were present: (1) The person experienced, witnessed, or was confronted with an event or events that involve actual or threatened death or serious injury, or a threat to the physical integrity of self or others, (2) The person’s response involved intense fear, helplessness, or horror.” Symptoms vary from patient to patient and can include recurrent and intrusive recollections of the event, recurrent dreams of the event and avoidance of stimuli associated with the event. Additional symptoms can include difficulty falling or staying asleep, irritability or outbursts of anger, difficulty concentrating, hypervigilance, and an exaggerated startle response. In the case of women the most frequent precipitating event is rape or physical assault whereas for most men it is a combat-related event (154).

The general observations made at the beginning of Section “Additional Assessments Recommended for Studies Investigating Traumatic Brain Injury and Specific Neuropsychiatric Disorders” concerning the assessment of psychiatric disorders following TBI apply with particular force when considering the assessment of PTSD. After reviewing the prior research on factor analysis of PTSD symptoms, Smith et al. (155) concluded, “There is thus reason to question whether PTSD is best considered to be a theoretically coherent psychological entity. Clearly, identical PTSD symptom counts can refer to different symptom pictures. It may not be in patients’ best interests to assign them a diagnosis that lacks clear meaning.” Nonetheless, the investigation of PTSD is now the focus of a major research effort.

The assessment recommendations presented in this paper are specifically directed to ERP studies in a TBI-positive population. Therefore, an operational question must be addressed: can PTSD occur as the result of a TBI? In 1996 Boake (160) wrote “Yet the preponderance of available evidence suggests that PTSD is not a major problem in the brain injured population. The traditional view of the relationship between brain injury and PTSD is that these disorders do not co-occur because they are incompatible.” Similarly, Bontke (156) reported that “At this point I can only claim to have seen one patient out of 2000 in the last 9 years with the dual diagnosis of PTSD and a mild TBI.” Sbordone and Liter (157) examined 28 patients with PCS and 42 with PTSD. The first author interviewed each patient individually for 2–3 h and asked them to describe their symptoms. In contrast to the patients diagnosed with PTSD, none of the mild TBI patients reported intrusive recollections of the traumatic event, nightmares. hypervigilance, phobic reactions, exaggerated startle reactions, or distress when asked to describe the traumatic event. Sbordone and Liter concluded that mild TBI and PTSD are incompatible.

This conclusion was challenged, however, by Bryant (158) and more recently in Bryant (159) who identified two critical methodological flaws in the Sbordone and Liter study: the interviewer was not blind to the status of each patient and standardized measures of PTSD were not used. Indeed, he directed attention to processes characteristic of PTSD following TBI including “implicit processing, biologically mediated fear conditioning and reconstruction of trauma events,” and ended his review by concluding that TBI and PTSD can co-exist. Consistent with Bryant’s (158) conclusion, PTSD has been reported in TBI populations (Table 8). Although it has been asserted that PTSD is highly improbable if not impossible in cases of brain injury when the patient does not have a memory of the injury event (156, 160), Joseph and Masterson (161) have argued that PTSD and TBI can co-occur either through a subconscious (i.e., implicit) level or through social reconstruction. Gil et al. (162) found that while explicit memory of the traumatizing event was a strong predictor of PTSD 6 months after a mild TBI, it was not an absolute requirement. Of the 55 participants with a memory of the traumatic event, 23% presented PTSD, while 6% of the patients who had no memory of the traumatic event met full diagnostic criteria for PTSD.

**Table 8:** **Incidence of PTSD in a TBI population**.

Source	Reported incidence of PTSD in a post-TBI population
Grigsby and Kaye (388)	36% *N* = 107
Rattok and Ross (389)	20% *N* = 40
Ohry et al. (390)	33% *N* = 24
Max et al. (391)	4% *N* = 46
Hickling et al. (172)	36% *N* = 107
Hibbard et al. (124)	19% *N* = 100
Harvey and Bryant (392, 462)	14% *N* = 79
Bryant et al. (393)	27% *N* = 96
Mayou et al. (394)	48% *N* = 261
Glaesser et al. (395)	27% loc less than 1 h, N = 15, 3% loc greater than 12 h *N* = 31
Gil et al. (162)	14% *N* = 120
Sumpter and McMillan (175)	*N* = 34, 59% via PDS, 44% via IES, 18% via CAPS, 3% (*N* = 1) via CAPS + Clinical Judgment
Hoge et al. (163)	43.9% TBI with loc, *N* = 124, 27.3% TBI with altered mental status, *N* = 260
Vanderploeg et al. (164)	Vietnam era veterans, 32.3% at discharge *N* = 155, 46.0% PTSD resolved
Zatzick et al. (165)	22.7% mild TBI, *N* = 406, 18.8% moderate TBI, *N* = 358, 16.8% severe TBI, *N* = 592
Bryant et al. (396)	12.7% *N* = 377
Wall (397)	Literature review 20 studies, 0.02–26%
Taylor et al. (398)	73%, *N* = 327,388 Veterans using VHA services
Bryan et al. (399)	32.67% *N* = 135
MacGregor et al. (400)	26.2% mTBI with LOC *N* = 103, 24.0% mTBI w/o LOC *N* = 150
Bazarian et al. (401)	17% *N* = 52

The more recent literature (163–165) convincingly argues that TBI and PTSD can indeed be comorbid. The question of neurological mechanism has been addressed by Gil et al. (162) who suggested that “One possible mechanism by which these results could be explained is that emotionally charged traumatic memories are initially processed with brain circuits that bypass cortical structures and are mediated primarily through the amygdale, resulting in the formation of implicit (unconscious) memories (166–168). In addition, the stress-induced secretion of glucocorticosteroids, which have been shown to impair hippocampal functioning, may disrupt the formation of explicit memory (169).” Further understanding of the neurological basis of a relationship between PTSD and TBI has been found by MacDonald et al. (170). Using diffusion tensor imaging to examine blast-induced TBI patients, these investigators found abnormalities in cingulum bundles, in right orbitofrontal white matter and in the middle cerebellar peduncles. Asymmetrically altered integrity of the cingulum bundle is associated with PTSD (171) and alteration in the right orbitofrontal cortex has been observed longitudinally in cancer patients who present PTSD. Like Gil et al. Zatzick et al. (165) found that individuals who had suffered a mild to moderate TBI were more likely to experience PTSD than were those who experienced a severe TBI.

All participants in the TBI/PTSD incompatibility debate agree that the assessment of PTSD in a post-TBI population presents formidable challenges, and a large number of diagnostic instruments have been proposed to address these challenges (Table 9). Hickling et al. (172) found that patients with PTSD could be misdiagnosed with TBI, and McMillan (173) found that individuals with TBI could be misdiagnosed with PTSD. These misdiagnoses could have resulted from differences in the diagnostic procedures used. For example, Harvey et al. (174) found that the use of standardized self-report questionnaires (seven different instruments were used in the studies reviewed) resulted in a high incidence of PTSD diagnoses in a post-TBI population while clinical interviews resulted in a low incidence. Similar results were reported in a study by Sumpter and McMillan (175) of 34 patients with severe TBI (post-traumatic amnesia greater than 1 day) whose PTSD symptoms were assessed using two self-report questionnaires, the Post-Traumatic Diagnostic Scale (176) and the Impact of Events Scale ((177) with the (178) cut-off score of 25 as the criterion for PTSD), and a structured clinical interview, the Clinician Administered PTSD Scale (CAPS, (179)). They found that 59% of the participants met criterion for PTSD using the Post-Traumatic Diagnostic Scale and 49% of the participants met criterion for PTSD using the Impact of Events Scale. However, very different incidence rates were obtained with the Clinician Administered PTSD Scale (179). In the “with judgment” variant, a symptom is scored if it is present and the administering clinician concludes that the symptom is related to the traumatic event. In the 2005 Sumpter and McMillan study, 18% of the participants met the CAPS PTSD diagnostic criterion based on symptom presence. If the additional requirement of clinical attribution of a symptom to the traumatic event was introduced, 3% (*N* = 1) of the sample met the diagnostic criterion. This should be compared with the 59% value obtained with the Post-Trauma Diagnostic Scale and the 49% obtained with the Impact of Event Scale with the same patient sample.

**Table 9:** **Instruments used in the assessment of PTSD**.

Instrument	Reference
Clinician administered PTSD scale	Blake et al. (179, 182, 183)
Subscales:
Re-experiencing (items 1–5)	
Avoidance (items 6–12)	
Hyperarousal (items 13–17)	
Davidson trauma scale (DTS)	Davidson et al. (402, 403)
Impact of event scale	Horowitz et al. (177), Weiss and Marmar (404)
M3 checklist	Gaynes et al. (405)
Penn inventory for posttraumatic stress disorder	Hammarberg (406)
Posttraumatic stress disorder interview-I	Watson et al. (407)
Posttraumatic diagnostic scale	Foa et al. (176, 408, 409)
Primary care PTSD screen PC-PTSD	Prins et al. (410)
PTSD checklist PCL military, civilian and specific incident additions	Weathers et al. (187), Blanchard et al. (190), Bliese et al. (411)
PTSD inventory	Solomon et al. (412)
PTSD module of the composite international diagnostic interview	Peters et al. (413)
Stanford acute stress reaction questionnaire SASRQ	Cardena et al. (414)
Trauma screening questionnaire	Brewin et al. (415)
Trauma symptom inventory, three validity scales (response level, atypical response, inconsistent Response), 10 clinical subscales	Briere (416), Berah (417)

In a subsequent paper, Sumpter and McMillan (180) analyzed the sources of diagnostic discrepancy between self-report questionnaires and the clinician administered structured interview. They cited the following possible causes. (1) Post-injury cognitive impairments caused errors in understanding. (2) Recurrent efforts to reconstruct memories lost during peri-trauma amnesia were scored as intrusive thoughts. (3) “Upsetting thoughts” that scored on the PTSD Diagnostic Scale were due to frustrations with post-injury physical limitations and not due to re-experiencing. (4) “Detachment” which scored on the questionnaire was also due, in some instances, to disability-dependent social isolation and not to a psychological consequence of the injury event. Sumpter and McMillan explicitly stated that their results do not indicate that PTSD cannot occur after severe TBI. They also noted that the observed diagnostic discrepancy may not be replicated in a mild TBI population. The results demonstrating the limitations of self-report questionnaires for assessing PTSD after TBI that were reported by Sumpter and McMillan are consistent with the conclusions of Sbordone and Ruff (181) and the recommendation of Bryant (158) who concluded: “Accordingly assessment for PTSD following TBI should not rely excessively on the client’s capacity to report relevant symptoms.” In the Sumpter and McMillan study, the self-report questionnaires did not, however, produce false negative assessments. Sumpter and McMillan therefore suggest that these questionnaires can be used as a first screen for PTSD following brain injury which is followed by a more demanding clinician administered instrument, for example the CAPS.

The Clinician Administered PTSD Scale CAPS, Blake et al. (179, 182, 183) is a structured interview containing 17 items scoring DSM criteria for PTSD. Frequency (0 = never, 1 = once a week, 2 = once or twice a week, 3 = several times a week, 4 = daily or almost every day) and intensity (0 = none, 1 = mild, 2 = moderate, 3 = severe, 4 = extreme) are scored for each item. Aggregate scores are produced by adding the frequency and intensity scores. Scores for a single item can therefore range from 0 to 8, and the aggregate score for the entire assessment can range from 0 to 136. Using signal detection theory Shalev et al. (184) determined that a CAPS score of 40 yielded 93% sensitivity and 80% specificity. The total score can be re-expressed as three subscales: re-experiencing (Items 1 to 5), Avoidance (Items 6–12), and Hyperarousal (Items 13–17). A factor analysis by King et al. (185) identified four correlated but distinct factors: re-experiencing, effortful avoidance, emotional numbing, and hyperarousal. Following Sumpter and McMillan (175), one can generate two CAPS scores, one without judgment (the symptom is present) and one with judgment (the symptom is present and is deemed to be a consequence of the traumatic incident). The instrument also provides five global scales: (1) Impact on Social Functioning, (2) Impact on Occupational Function, (3) Global Validity, (4) Global Severity, and (5) Global Improvement.

The National Center for PTSD (United States Department of Veterans Affairs) advises that “the CAPS was designed to be administered by clinicians and clinical researchers who have a working knowledge of PTSD, but can also be administered by appropriately trained paraprofessionals.” The CAPS training CD-ROM can be ordered from the National Technical Information Service. The International Association of Trauma Professionals provides certification for administration, scoring, and interpretation of the CAPS. The CAPS-CA (186) is a version of CAPS that is appropriate for children and adolescents.

The PTSD Checklist [PCL, (187)] has three versions. The PCL-M measures responses to stressful military experiences. The PCL-C is directed to civilians and assesses symptoms in relation to generic stressful occurrences, and the PCL-S is used in studies of symptoms relating to a specific stressful experience. There are minimal differences between the three versions. They all have 17 questions scored on a scale of 1 = “Not at All” to 5 = “Extremely.” A higher score indicates a greater symptom burden. The questions are divided into three groups designated Group B, C, and D. Group B questions (Questions 1–5) assess intrusive thoughts, disturbing dreams, re-experiencing, and physical (autonomic) responses to memory of the stressful experience. Group C questions (Questions 6–12) score avoidance, memory deficits, psychological numbing, and social isolation. Group D questions (Questions 13–17) evaluate sleep, labile affect, and concentration difficulties. Patients will meet DSM-IV diagnostic criteria for PTSD if they have a moderate or severe response (scores 3–5) for at least one Group B question, three or more Group C questions and two or more Group D questions. The National Center for PTSD (188) proposes cut scores of 30–35 for the general population (civilian primary care, Department of Defense screening and general population screening), 36–44 for specialized medical clinics (TBI clinics, pain clinics, VA primary care) and 45–50 for VA or civilian specialty mental health clinics). Monson et al. (189) concluded that when used longitudinally a change of 5–10 points is reliable (that is, it is not due to chance), and a change of 10–20 points is clinically significant.

Several investigators have examined the psychometric properties of the PCL. Blanchard et al. (190) studied motor vehicle accident patients and sexual assault patients and used the CAPS diagnosis as the dispositive metric. They found that a PCL cutoff of 44 had a sensitivity of 0.94 and a specificity of 0.86. Norris and Hamblen (191) studied Viet Nam era veterans. The internal consistency of the total score was 0.97. The internal consistency of subscales ranged from 0.92 to 0.93. The 2-day test-retest reliability was 0.46. The correlation with the Mississippi Scale of Combat-Related PTSD was 0.93. The correlation with the PK Scale of the MMPI (Minnesota Multiphasic Personality Inventory) was 0.77, and the correlation with the impact of Events Scale was 0.90. In a study in which a DSM-derived Structured Clinical Interview for Diagnosis was used as the defining metric, the PCL has a sensitivity of 0.82 and a specificity of 0.83 when a PCL score of 50 was used as the cut-off score.

Wilkins et al. (192) reviewed 72 studies using the PCL that were conducted between 1993 and 2010. In summary, they found that the PCL is psychometrically sound. They cite ease of administration as one of its strengths. Two weaknesses were identified. They suggested that the PCL may be above the reading level of some participants. Additionally, they found that the PCL may over-estimate the prevalence of PTSD. This observation is consistent with the results of Ruggiero et al. (193). Ruggiero et al. used both the National Women’s Study PTSD module (NWS-PTSD) and the PCL to assess 233 New York City area residents 4 months after the 9/11 attacks. In this sample, the prevalence was 1.7% for the NWS-PTSD and 4.1% for the PCL.

Administration of the CAPS requires 45–60 min. For this reason, it is not recommended for an initial PTSD screening in studies where PTSD is not the primary focus of the investigation. Administration of the PTSD Checklist requires approximately 5 min. We follow the proposal of McDonald and Calhoun (194) in recommending that the PCL be used as a screening test to be followed with a second-tier diagnostic evaluation. We recommend using the CAPS in this second-tier evaluation with the Shalev et al. cut-off score of 40.

### Assessment of anxiety

We consider here anxiety disorders following TBI that do not meet PTSD diagnostic criteria. TBI is a significant risk factor for anxiety disorders. Epstein and Ursano (195) reviewed 11 studies with a total post-TBI clinical population of 1119 and reported an aggregate incidence of 29%. As noted, however, by Hiott and Labbate (196) some of the studies summarized in Epstein and Ursano predate the publication of DSM-III criteria. Interpretation is, therefore, difficult. In a more recent review, Warden and Labbate (197) cited the following incidence of anxiety disorders following TBI: generalized anxiety disorder 8–24%, panic disorder 2–7%, obsessive-compulsive disorder 1–2%, and specific phobias (especially driving) less than 25%. For the specific diagnosis of generalized anxiety disorder following TBI (see Table 10), Hiott and Labbate reviewed four studies and found a cumulative incidence of 10.2% (26 of 254). This should be compared against the lifetime prevalence rate of generalized anxiety disorder in the general population of 5.1% reported by Kessler et al. (198) and approximately 6% reported by Ritter et al. (199). However, determination of incidence rates is complicated by symptom overlap between disorders and by comorbidities. Jorge et al. (104) found that two-thirds of the post-TBI patients in their sample presenting major depression also met the diagnostic criteria for generalized anxiety disorder.

**Table 10:** **Incidence of generalized anxiety disorder in a TBI population**.

Source	Reported incidence of generalized anxiety disorder in a TBI population
Jorge et al. (103)	11% *N* = 66
Fann et al. (377)	24% *N* = 50
van Reekum et al. (418)	22% *N* = 10
Hibbard et al. (124)	9% *N* = 100
Deb et al. (419)	2.5% *N* = 120

Shear et al. (200) discuss several standardized instruments for assessing anxiety disorders. No single instrument has emerged as the preferred choice for studies of TBI-related anxiety disorders Because it has been used in a number of studies of anxiety following TBI (201–203) we recommend the Beck Anxiety Inventory (204, 458) for a rapid assessment of general anxiety and mixed anxiety disorders. Instruments for assessing specific anxiety disorders, such as panic disorder, social phobia, obsessive-compulsive disorder, and generalized anxiety disorder, are also described in Shear et al. (200).

### Assessment of psychotic disorders

Psychotic disorders can follow TBI (205–207), but there is considerable uncertainty about the frequency of post-injury psychosis. Davison and Bagley (208) [as cited by (209)] found the 10- to 20-year incidence of psychosis following TBI to be two to three times higher than in the general population. Achte et al. (210) reviewed cases of 3000 combat veterans who had suffered moderate to severe brain injury and found that 750 (25%) of these patients displayed psychotic symptoms. Paranoid schizophrenia and paranoid schizophreniform psychosis developed earlier (23% within 1 year) than did delusional psychosis (4%). Delusional psychosis lasted less than a year in 28% of the cases and more than 5 years in 40% of the cases. Thomsen (211) followed 40 TBI patients with severe blunt head trauma. Twenty percent (*N* = 8) developed psychotic disorders. Early onset of psychotic symptoms occurred in two patients (3 and 5 months post-injury), and six patients presented delayed-onset psychosis (1–6 years post-injury). For comparison, Perälä et al. (212). reported lifetime prevalence in a general population to be 0.87% for schizophrenia, 0.32% for schizoaffective disorders, 0.07% for schizophreniform disorder, and 0.18% for delusional disorder.

The attribution of psychosis to brain injury is complicated not only by the delayed onset of psychotic symptoms (114–116), but also by the lack of uniformity in the occurrence of TBI in the population. Malaspina et al. (213) found that the “rate of TBI was significantly higher for those with a diagnosis of schizophrenia, bipolar disorder, and depression than for those with no mental illness.” It therefore seems possible that in some instances brain injuries that evolved to psychosis were sustained by individuals who either had premorbid histories of psychotic disorders or were at increased risk of developing a psychosis prior to the injury. This later speculation is consistent with the finding of Sachdev et al. (116) who found that, along with the duration of LOC, a family history of psychotic illness was the best predictor of psychosis following TBI. An argument for a causative role of TBI in some instances of psychotic illness can nonetheless be made. Wilcox and Nasrallah (214) reviewed medical histories of 200 schizophrenic patients, 203 depressed patients, 122 manic patients, and 134 surgical controls. Histories were examined to determine if there was a history of head injury before the age of 10 (an age well before the appearance of psychotic symptoms) severe enough to require medical attention or a LOC due to head injury. Schizophrenics had a significantly greater frequency of TBI than did depressives, manics or surgical controls Wilcox and Nasrallah argued that because the head injuries occurred before the age of 10, deficits in premorbid functioning did not predispose schizophrenics to head injuries, suggesting that head injury may have a causative role in some presentations of schizophrenia. Arciniegas et al. (205) noted that the age of onset of psychosis following TBI does not follow the typical pattern of 18–25 years for males and 25–30 years for females [citing (115, 116, 215)]. Arciniegas et al. (205) also noted that at least in some instances, symptom types include comorbid seizure disorders and associated cognitive impairments which are not typical in a primary psychotic disorder. This further argues for a role of TBI in the etiology of post-injury psychosis in some patients, support for which is provided in a meta-analysis by Molloy et al. (216) who found a significant association between TBI and schizophrenia. Additionally, estimates they derived from family studies were higher than those from cohort case-control studies by a factor of almost two. Consequently Molloy et al. concluded “this meta-analysis supports an increased risk of schizophrenia following TBI with a larger effect in those with a genetic predisposition to psychosis.”

Symptom-Checklist-90-R includes paranoid ideation and psychoticism subscales. For most ERP studies with TBI patients, this will be sufficient for studies that do not have post-TBI psychotic disorders as a primary focus. For research studies where psychotic disorders are a central concern, we recommend the clinician administered Scale for the Assessment of Positive Symptoms [SAPS, (217)] and the Scale for the Assessment of Negative Symptoms [SANS, (218)]. The SAPS has 30 items and four domains covering (1) hallucinations, (2) delusions, (3) bizarre behavior, and (4) positive formal thought disorders (incoherence, distractible speech, clanging). The SANS has 20 items covering five domains: (1) affective flattening or blunting, (2) alogia, (3) avolition-apathy, (4) anhedonia-asociality, and (5) attention. Participant burden is significant. Administration of each scale requires approximately 30 min. It should be noted that clinician training is required in order to achieve reliable ratings.

### Sleep disorders

In part because of the social acceptability of symptoms associated with sleep disorders, they are among the most frequently endorsed symptoms following TBI (219–221). However, evaluation of a causal role of TBI in disturbed sleep is difficult for several reasons. First, the sleep disorder may be pre-existing. Moreover, quantitative characterization of the epidemiology of sleep disorders in the general population, which provides an essential comparator to the TBI population, is complicated by a lack of uniformity in reporting criteria. For example, Hochstrasser (222) reported a prevalence of sleep disturbance in the general population of 26% with a 13% prevalence of moderate to severe disturbance. Ford and Kamerow (223) found that 10% of the general population endorses symptoms of insomnia, and Rosekind (224) reported 30%. An additional factor complicates the evaluation of sleep disturbances following TBI. As we previously reviewed, depression is a common sequel to TBI, and as reviewed by Masoodi and Jiva (225), sleep disturbances are a frequent element in depressive illness. This TBI-depression-sleep disorder confound is documented in Fichtenberg et al. (226) who found a significant correlation between insomnia and depression, as documented by the BDI, in a TBI population. It is estimated that 50–80% of patients with a psychiatric disorder present sleep disturbances that can be attributed to the underlying psychiatric disorder. Further, the pattern of sleep disturbances following TBI is highly varied from patient to patient thereby complicating epidemiological study. In Table 11 we have listed the incidence of sleep disorders among TBI patients as reported across several studies.

**Table 11:** **Incidence of sleep disorders in a TBI population**.

Source	Reported incidence of sleep disorders in a TBI population
Dikmen et al. (420)	40% *N* = 20
Cohen et al. (421)	73% recent injury, *N* = 22, 52%. Discharged patients, *N* = 77
Beetar et al. (422)	56% *N* = 202
Perlis et al. (423)	65% *N* = 39
Clinchot et al. (424)	50% *N* = 130
Fichtenberg et al. (425)	30% *N* = 50
Ouellet et al. (426)	29% *N* = 452
Korinthenberg et al. (427)	10% *N* = 98
Parcell et al. (428)	80% *N* = 63
Lundin et al. (429)	49% *N* = 122
Castriotta et al. (430)	46% *N* = 87
Makley et al. (431)	68% *N* = 31

Instruments for assessing sleep disorders have been reviewed by Benca and Lichstein (227) who began their review by observing that the definitive assessment of sleep disorders requires a polysomnographic study. With this understanding, we recommend the Pittsburgh Sleep Quality Index [PSQI, (228)]. It is the most comprehensive of the available instruments, and it has been validated with TBI patients. The internal consistency of the PSQI is good [Cronbach α > 0.8; (228, 230, 231, 459)]. The test-retest reliability, as quantified by the Pearson linear correlation coefficient is 0.85 for the global score and 0.65–0.84 for the component scores. The validity of the instrument was assessed by its ability to discriminate between different populations. In the Buysse et al. (228) study, the two groups were good and poor sleepers. Using a global score cutoff of five gave a sensitivity of 89.6% and a specificity of 86.5%. Backhaus et al. (230) compared healthy controls and insomnia patients. Using the same cut-off score, they observed a sensitivity of 98.7% and a specificity of 84.4%. Administration of the PSQI requires 5–10 min. It contains 19 self-rated multiple choice questions and four write-in questions (typical bedtime, typical wakeup time, sleep latency, sleep duration). An additional five multiple choice questions are to be answered by a bed partner or roommate. They are not used in scoring. The instrument generates scores for seven component scales. A global score greater than five is deemed to indicate significant dysfunction. There are no cut-off scores for component scales. The seven component scales are Subjective Sleep Quality, Sleep Latency, Sleep Duration, Habitual Sleep Efficiency, Sleep Disturbances, Use of Sleep Medications, and Daytime Dysfunction.

Fichtenberg et al. (232) conducted a validation study of the PSQI in a TBI population. Data taken from sleep diaries and interviews were used to classify 91 consecutive patients admitted to an outpatient neurorehabilitation program as insomnia or non-insomnia based on DSM-IV criteria. Patients taking sleep medications were excluded from the study. Using a PSQI global score of>8 as the cutoff, the sample was classified with 93% sensitivity and 100% specificity. Classification was also determined using the component scores. The criterion of sleep onset>30 min more than twice a week gave an accurate classification for 92% of the sample. Sleep duration<6.5 h more than twice a week had 82% accuracy, and sleep efficiency (the amount of time asleep divided by the amount of time in bed)<85% more than twice a week gave an accurate classification in 74% of the cases. A further classification was constructed by requiring two or more of the component score criteria described above to be satisfied. This procedure had a sensitivity of 100% and a specificity of 91%. For purposes of ERP studies it is typically not necessary to make a dichotomous insomnia/non-insomnia distinction. In studies where sleep quality is a measure of interest we recommend reporting both the global score and the seven component scores.

### Assessment of suicidal ideation

Traumatic brain injury is a risk factor for suicide (233–236), suicide attempts (237, 238), and suicidal ideation (106, 239). The most exhaustive investigation to date of the relationship between TBI and suicide was conducted by Brenner et al. (240) who studied individuals receiving Veterans Health Administration services between 2001 and 2006. Patients with a history of TBI (*N* = 49,626) were compared with no history of brain injury (*N* = 389,053). Models were adjusted for demographic and psychiatric covariates. Veterans in the TBI-positive group were 1.55 times more likely to die by suicide than veterans in the TBI-negative group. The relationship between TBI and suicide is particularly pressing for the military because of the high incidence of TBI and suicide in the military population. Approximately 64% of OEF/OIF personnel wounded on active duty are wounded by blast events (241) indicating the presence of a large at-risk population in the military. The increased incidence of TBI in the military coincides with an increase in Army suicide rates from 9 per 100,000 in 2001 to 22 per 100,000 in 2009 (242). This should be compared with a global incidence of 16 per 100,000 per year (243).

Several instruments for assessing suicidal behavior and suicidal ideation are available (244). The Columbia Suicide History Form (244, 245) documents previous suicide attempts, and the Suicide Intent Scale (246) assesses the intensity of an attempter’s wish to die at the time of the attempt. Neither scale is appropriate for evaluating suicidal ideation in individuals who do not have a history of attempted suicide. The risk of suicide following TBI has been assessed by Léon-Carrion et al. (247) using response Rorschach profiles evaluated with Exner’s (248) scoring system to assess the risk of suicide, but this method requires expertise that is not typically available in an ERP laboratory. As an alternative, Tsaousides et al. (249) used the suicide related questions of the Beck Depression Inventory in their study of suicidal ideation following TBI. We suggest that either the BDI or the SCL-90-R, which asks about thoughts of ending life, is adequate for studies where suicide risk is not a major focus. For studies where suicidal ideation is a matter of specific interest, we join with Dennis et al. (250) in recommending the Beck Hopelessness Scale (251, 252) that was also used by Simpson and Tate (238) and Simpson et al. (253) in studies of suicide prevention after TBI.

The Beck Hopelessness Scale (252) consists of 20 true/false questions and can be self-administered or administered verbally by a clinician. A global score from 0 to 20 is formed by summing individual items. Beck and Steer proposed the following classification: 0–3 minimal, 4–8 mild, 9–14 moderate, 15–20 severe. It has good internal consistency, Pearson *r* = 0.82–0.93 for different populations and test-retest reliability at 1 week of *r* = 0.69, and at 6 weeks of *r* = 0.66 (252). Moreover, the validity of the measure was assessed by comparing the Beck Hopelessness Scale with measures of depression (252, 254). Nekanda-Trepka et al. (254) found that the Hopelessness Score was positively correlated with the aggregate BDI score (*r* = 0.47, *p* = 0.001). In a prospective study of 1958 psychiatric outpatients, Beck et al. (255) found that individuals with a global score of greater than or equal to 9 were 11 times more likely to commit suicide than those with a lower score. This cut-off score identified 16 of the 17 individuals in the sample who committed suicide. It should be noted, however, that because the incidence of completed suicide is low, this criterion yields a high incidence of false positive evaluations, 59.0%. Keller and Wolfersdorf (256) followed 61 depressed patients for 1 year. During this period there were eight suicide attempts and two completed suicides. A cut-off score of eight on the Hopelessness Scale successfully identified 90% of the suicidal actions. These investigators also found a high incidence of false positives.

### Assessment of alcohol abuse

The association between alcohol and TBI is a complicated one because alcohol use is often a causative factor in civilian brain injury. Kraus et al. (257) found that 56% of adult civilians with a brain injury had a positive blood alcohol concentration at the time of injury, of whom 49% had a blood alcohol concentration in excess of the legal limit. Similarly, Sparedo and Gill (258) found that 67% of TBI patients tested positive for alcohol and 51% were intoxicated when the injury occurred. That these levels may be associated with pre-injury alcohol abuse is suggested by Kreutzer et al. (259) who reported a high incidence of heavy alcohol consumption both before and after injury. That a history of alcohol use or abuse is strongly associated with a higher risk of suffering a TBI is suggested by Hillbom and Holm (260) who found that the overall incidence of head injury in alcoholics is two to four times the incidence in the general population. Using the Quantity-Frequency-Variability Index (261, 262) to characterize alcohol consumption, Horner et al. (263) found that at 1 year post-injury 15.4% of the TBI sample were heavy drinkers and 14.3% were moderate drinkers. Bombardier et al. (264) found that drinking decreased following injury, but approximately 25% of their sample reported heavy drinking 1 year post-injury. Ponsford et al. (265) found that pre-injury TBI and appropriately matched control populations showed similar alcohol consumption. In the pre-injury TBI group, 31.4% used alcohol at a hazardous level, and 29.3% of the controls used alcohol at a hazardous level (In this study hazardous use was defined as a score greater or equal to eight on the Alcohol Use Disorder Identification Test. This instrument is discussed presently.). As did Bombardier et al. Ponsford et al. found that alcohol abuse declined post-injury, but it subsequently increased with 25.4% of the TBI group drinking at hazardous levels 2 years post-injury. In a 30-year follow-up study using DSM-IV criteria for alcohol dependence and alcohol abuse, Koponen et al. (266) found that 11.7% of their sample either abused alcohol or were alcohol dependent. This sample had a pre-injury prevalence of 8.3%.

Alcohol use following head injury is higher than in the general population. In part this is to be expected since alcohol misuse may have been present prior to injury, but post-injury factors are also important. Reilly et al. (267) identified four psychosocial factors that increase the risk of alcohol abuse after TBI: (1) increased discretionary time and boredom, (2) increased enabling from family and friends, (3) uncertainty over the ability to return to work or to function effectively at work, and (4) physical limitations and post-traumatic mood change. Horner et al. (263) found six risk factors for heavy drinking following TBI: (1) gender, (2) young age, (3) history of abuse prior to TBI, (4) diagnosis of depression since TBI, (5) fair/moderate mental health, and (6) better physical functioning. Recidivism following completion of alcohol rehabilitation is high in the post-TBI population. Sparedo and Gill (258) found that 54% of patients who completed alcohol rehabilitation returned to alcohol. Of the remaining 46%, post-injury seizure disorders or placement into long-term supervised living were significant factors in maintaining abstinence.

Martino et al. (268) listed 14 instruments for assessing alcohol abuse and for planning and monitoring treatment for alcohol abuse. Of these, we recommend the Alcohol Use Disorders Identification Test [AUDIT (269)]. Martino et al. cited AUDIT’s slightly better psychometric performance as quantified by internal consistency, test-retest reliability, and validity. In a review of 13 psychometric studies of AUDIT, Reinert and Allen (270) concluded that AUDIT is comparable and typically superior to other self-report screening measures. AUDIT measures a continuum of alcohol use and has proven to be of value in characterizing this continuum. It may therefore be useful in identifying clinically significant alcohol misuse at an early stage of drinking before it has reached the level of alcohol dependence.

AUDIT consists of 10 questions scored from 0 = “never” to 4 = “daily or almost daily.” A global score is obtained by summing the scores of individual test items. Babor et al. (269) indicate that for men less than 65 years old, a score greater than or equal to eight indicates hazardous and harmful alcohol use. For men over 65 and all women, they recommend a cut-off score for hazardous use of seven. A score greater than or equal to 20 “clearly warrants further diagnostic evaluations for alcohol consumption.” The instrument has a high internal consistency (271, 272). AUDIT is also consistent with other instruments that assess alcohol use. Bohn et al. (273) found a correlation between AUDIT and the Michigan Alcohol Screening Test [MAST, (274)] of=0.88. The correlation with the four question CAGE Questionnaire (275) is *r* = 0.78 (272). The instrument has also been shown to be a valid indicator of alcohol impact on global life functioning. Claussen and Aasland (276) found that the probability of being unemployed over a 2 year period was 1.6 times higher for individuals with an AUDIT score greater than or equal to eight. Conigrave et al. (277) found that AUDIT scores predict future occurrences of physical disorders. The AUDIT score is not affected by question ordering or the wording of questions (278). Several groups have investigated the test-retest reliability of AUDIT reporting values of *r* = 0.92 ((279), university students, 2 week interval), *r* = 0.81 (280) (primary care patients, 6 week interval), and *r* = 0.64 (281) (primary care patients selected for alcohol treatment, 2 week interval). Further results of validity and reliability testing of AUDIT are given in Reinert and Allen (270).

### Substance abuse

Most of the literature examining the relationship between substance abuse and TBI limits the discussion to alcohol. The literature that is available does, however, establish that as in the case of alcohol, the use of illicit drugs is a risk factor for sustaining TBI, and a history of pre-injury substance abuse is correlated with increased disability, delayed recovery, and poor outcome [reviewed in (282–285)]. Based on a review of the literature, Graham and Cardon reported that substance abuse rates decline following TBI, including mild TBI. Based on our non-systematic review of the literature, we concluded that the use of illicit drugs following TBI in individuals who did not have prior history of drug use is unusual.

We recommend the Drug Abuse Screening Test [DAST, (286)] for assessing drug abuse or dependence on psychoactive drugs other than alcohol. The DAST can be self-administered or administered in an interview by a clinician. It consists of a series of yes/no questions with zero being scored for no and one being scored for yes. A global score is constructed by summing the responses to individual items. There are two versions, 20 question and 28 questions. A global score greater or equal to five obtained with the 28 question version indicates that a drug disorder is probable. The instrument is consistent having a Cronbach α of 0.92 for individuals with a substance abuse disorder and of 0.94 for general psychiatric admissions (286). Using a DSM-III-R diagnosis by a psychiatrist as the reference standard, Gavin et al. (287) found that a cut-off score of 5 had a sensitivity of 0.96 and a specificity of 0.79.

## Cognitive Assessments

### Executive function

Executive functions are broadly defined as the integrative and organizing functions of cognition. In the Cicerone et al. (288) characterization, executive functions can be categorized into four domains: executive cognitive functions (planning and organization), behavioral self-regulation functions (emotional processing, understanding the consequences of behavior), activation regulating functions (decreased initiation), and metacognitive processes (self-awareness). All or some of these functions can be impaired following brain injury. Malloy and Grace (289) reviewed five instruments for assessing executive function: the Behavior Rating Inventory of Executive function [BRIEF, (290)], the Dysexecutive Questionnaire [DEX, (291)], the Frontal Behavioral Inventory [FBI, (292)], the Frontal Systems Behavior Scale [FrSBe (293, 294)], and the Iowa Rating Scales of Personality Change [IRSPC, (295)]. Of these, Malloy and Grace note that the BRIEF and the FrSBe have good reliability and large scale norms. The BRIEF, however, is a measure for pediatric populations and normative data are only available for children up to 18 years old. The FrSBe has normative data for 18–95 years. A further argument for using the FrSBe in studies of TBI is provided by Reid-Arndt et al. (296) who found that while neuropsychological tests of executive function did not help predict post-injury community integration, the FrSBe did predict important functional outcomes. Additionally, the Apathy Subscale of the FrSBe (described below) was investigated by Lane-Brown and Tate (297) who found that it was a reliable and valid measure of apathy following TBI. Therefore, we recommend using the FrSBe in ERP studies of TBI where executive function is topic of interest.

The Frontal System Behavior Scale (293) is a 46 item questionnaire with each item score on a scale of 1, “Almost Never” to 5 “Almost Always.” A high score indicates greater disability. It is composed of three subscales. Subscale A, 14 items, assesses apathy, and akinesia (anterior cingulate). Subscale D contains 15 items and evaluates disinhibition and emotional dysregulation (orbital frontal cortex). Scale E, 16 items, evaluates deficits in executive function (dorsolateral prefrontal cortex).

### Cognitive insight

Insight is a word of many meanings. We have considered two distinct capabilities, cognitive insight (an assessment of the patient’s self-understanding) and (ii) the ability to form novel insight-dependent associations. Both can be significantly impaired following a TBI. At present there are no procedures for constructing premorbid estimates of these measures. In the absence of pre-injury measurement, their use will be limited to post-injury longitudinal assessments. Their utility in this regard should not, however, be under-estimated.

Several instruments for evaluating insight [see Ref. (229)] have been published. These are directed to evaluating insight in psychotic patients. The Beck Cognitive Insight Scale [BCIS, (229)] serves that function but is more broadly constructed and is applicable to other populations. It is therefore recommended for use in TBI studies. The instrument assesses self-reflectiveness about unusual experiences, the capacity to correct erroneous judgments and certainty about mistaken judgments. It is self-administered and contains 15 items that are scored on a four-point scale with 0 = “Do Not Agree at All” to 3 = “Agree Completely.” There are two subscales. The self-reflectiveness scale has nine items and evaluates objectivity, reflectiveness, and openness to feedback. The self-certainty subscale has six items and assesses certainty about beliefs and conclusions. A cognitive insight score is calculated by subtracting the aggregate self-certainty score from the aggregate self-reflectiveness score. Factor analysis indicated that the two subscales are minimally correlated. Internal consistency was indicated by α scores of 0.68 (self-reflectiveness) and 0.60 (self-certainty). Beck et al. acknowledge that these values are less than the 0.7 value recommended by Nunnally (298) but note that the prior literature (299, 300) indicates that these values are acceptable for research purposes. Construct validity for inpatients diagnosed with schizophrenia or schizoaffective disorders was indicated by comparisons with the Scale to Assess Unawareness of Mental Disorders (301). Construct validity was further supported by a study by Granholm et al. (302) who found that changes scores in positive and negative symptoms in response to treatment for schizophrenia were significantly correlated with changes scores on the BCIS.

### Insight formation

Typically, assessment following TBI is based on tasks that are simple, undemanding tasks that can be accomplished in the absence of intellectual insight. Life is not simple. It is not undemanding, and it frequently demands insight formation. It is possible that a test of higher cognitive processes can identify significant injury-derived deficits in individuals who present normal results in standard assessments. Tasks based on the discovery of compound remote associates provide a means of testing the capacity for insight. Schooler et al. (303) have published the following definition of an insight problem. It is a problem that

“(a) is well within the competence of the average subject; (b) has a high probability of leading to an impasse, that is, a state in which the subject does not know what to do next; and (c) has a high probability of rewarding sustained effort with an ‘Aha’ experience in which the impasse is suddenly broken and insight in to the solution is rapidly attained.”

The remote associates test was introduced by Mednick (304) in studies of creativity. In the remote associates test, words from mutually distinct associative clusters are presented to the participant who must find a word that provides an associative mediating link between them. For example, the stimulus words rat, blue and cottage have the associative connection cheese. Success on this test has been shown to correlate with other tests of insight formation (305, 306). The test has also been used in investigations of psychopathology (307) (specifically subclinical predisposition to manic depression), in investigations of reactions to positive affect (308), and in research on self-esteem (309). The compound remote associate problem is a specific type of remote associate task (310). For example, in this task, three stem words (pine, crab, sauce) are presented simultaneously to the subject. The subject’s task is to find a single-word that can be used to form a compound word or phrase with all three test words (apple to give pineapple, crab apple, and applesauce). The previous example from Mednick’s remote associates test (rat, blue, cottage) would not satisfy the more restrictive criterion of a compound remote associate stimulus, but the stimulus triple board, blue, and cottage would meet the criterion. Bowden and Jung-Beeman (310) have constructed and tested 144 compound associate problems. Their documentation provides normative data regarding the percentage subjects solving the problem within a given time limit (2, 7, 15, or 30 s) and the average time-to-solution of those trials where the problem was solved.

Bowden and Jung-Beeman (310) list the following advantages that compound remote associate problems have over what they term “classical insight problems.”

“(1) They can be solved in a short time, so that many can be attempted in a single experimental session of 1 h or less. (2) They are simpler than classic insight problems, thus allowing better control of possible confounding variables. (3) They have single-word, unambiguous solutions, making scoring of responses easier. (4) They are physically compact, so that they can be presented in a small visual space or short time span.”

To this we would add that the test can be performed with a laptop computer. A sophisticated laboratory infrastructure is not required.

It should be noted that it is possible to incorporate a compound remote associate task in an experimental protocol that incorporates simultaneous neurophysiological measures (311, 312). Bowden and Jung-Beeman (313) and Jung-Beeman and Bowden (314) have used compound remote associates to investigate differential hemispheric contributions to problem solving and hemispheric contributions to the experience of a moment of insight when they are solved. The two phenomena, they argue, are distinct. In many cases, the subject solves the problem but does not have an experience of a punctate transition to the solution, what Bowden and Jung-Beeman refer to as the “Aha! experience.” In a modification of the basic experimental procedure that includes electroencephalography and an fMRI study, the three stem words were presented to the left or to the right visual hemifield. Bowden and Jung-Beeman concluded that semantic activation of the right hemisphere may help solve insight problems (313), and that the right hemisphere maintains solution-related activation for yet to be solved problems (314). Bowden and Jung-Beeman (315) subsequently found that the insight experience correlates with solution activation in the right hemisphere (see Table 12).

**Table 12:** **Instruments recommended for cognitive assessments**.

Domain assessed	Instrument	Reference
Executive function	Frontal systems behavior scale (FrSBe)	Grace and Malloy (293), Stout et al. (294)
Cognitive insight	Beck cognitive insight scale (BCIS)	Beck et al. (229)
Insight formation	Remote associates test	Bowden and Jung-Beeman (310)

## Sociological Assessments

### Socio-economic status

As outlined in preceding sections some patients who sustain a TBI may be asymptomatic or minimally symptomatic immediately following injury but subsequently present neuropsychiatric disorders. The ability of ERPs to identify individuals at risk of delayed-onset psychiatric presentations is now being investigated. Given the heterogeneity of this clinical population, however, it is not suggested that ERPs alone will provide a uniformly successful indicator. ERP data must be combined with other physiological measures including serum biomarkers and imaging studies. Sociological factors, for example socio-economic status, SES, should also be considered. Research has established a correlation between PTSD and depression following injury or traumatic experiences with socio-economic status (316–320). Evidence indicates that both possible causal relationships can occur, that is psychiatric disorders result in lower socio-economic status, but conversely low socio-economic status is a risk factor for psychiatric disorders after a traumatic event. Socio-economic status may therefore be an important complement to physiological measures in efforts to identify individuals at risk of delayed-onset psychiatric disorders following TBI. Additionally, it is important to control for socio-economic status when constructing matching participant groups in clinical studies. This can be especially relevant for ERP studies as correlations between differences in SES and differences in ERPs have been observed (321–323).

The assessment of socio-economic status is, however, exceptionally difficult, Braverman et al. (324) summarized the situation admirably in the title of their JAMA review, “Socio-economic status in health research: one size does not fit all.” While recognizing these difficulties, we recommend the Barratt Simplified Measure of Social Status [BSMSS, (325)] because of its simplicity and public domain availability. The aggregate score is computed from knowledge of the education and occupation of the participant, the participant’s spouse/partner, and the participant’s parents. Educational status is partitioned onto a seven-element scale, and occupations are classified into nine elements. The scale is derived from the widely used Hollingshead (326, 327) scale. Barrett made two significant modifications. First, the list of occupations reflects research updating occupational prestige ratings (328, 329). Second, the Barratt instrument incorporates scoring of parental and partner education and occupation. Adjustments are made in the scoring algorithm for participants who are not married/partnered and for participants who grew up in a single parent family. Parental scores are used for full time students.

The estimation of the socio-economic status of active duty military personnel is complicated. Past research which indicated that occupation is the best single indicator of SES (330) has no discriminatory power within this population since by definition they all have the same employer. In the case of military personnel, the procedure in Barratt for full time students should be used; namely the SES of the family of origin should be reported.

### Social support

Social support and patient perception of social support is a significant factor in the recovery from any illness or injury. A substantial body of literature indicates this is true of TBI. Perceived social support can be a significant predictor of neuropsychiatric disorders and post-injury community integration (331–333) (The relationship between social support and neuropsychiatric sequelae can, however, be complex. Leach et al. (334) found that effective use of problem solving and behavioral coping strategies by the family of a TBI patient correlated with reduced incidence of depressive illness, but perceived social support was not predictive of depression.).

The Multidimensional Scale of Perceived Social Support (335) measures subjective assessment of social support adequacy from three specific sources. Twelve items are scored on a scale from 1 (Very Strongly Disagree) to 7 (Very Strongly Agree). The instrument provides a total score and scores on three subscales (Significant Other, Family, Friends). In a study with undergraduates reliability was indicated by Cronbach alphas of 0.91 (Significant Other), 0.87 (Family), 0.85 (Friends), and 0.88 (Total Score). The test-retest reliability scores were 0.72 (Significant Other), 0.85 (Family), 0.75 (Friends), and 0.85 (Total Score). The construct validity was examined by comparisons with the Depression and the Anxiety subscores of the Hopkins Symptom Checklist [HSCL, a precursor of the SCL-90, (336)]. Zimet et al. hypothesized the perceived social support should be negatively related to depression and anxiety. Perceived support from family was inversely related to depression (*r* = −0.24, *p* < 0.01) and anxiety (*r* = −0.18, *p* < 0.01). Support from friends was inversely related to depression (*r* = −0.24, *p* < 0.01) as was support from significant others (*r* = −0.13, *p* < 0.05) and the aggregate score (*r* = −0.25, *p* < 0.01). These psychometric results were subsequently confirmed with other participant populations (337, 338).

### Quality of life

Bullinger et al. (339) have correctly observed that the report of family members should not be used as a proxy measure of the patient’s quality of life. When making this assessment, a distinction should be made between an assessment of the health-related quality of life and community integration. We consider here health-related quality of life. Community integration is considered in the next section.

Some investigators, for example Guilfoyle et al. (340) and Beseoglu et al. (341) have used the Short Form 36 (SF36) to assess health-related quality of life. While this measure is not specific to TBI, it is often an acceptable measure in TBI studies. A possible exception to this observation would be rehabilitation and treatment studies of TBI. In these studies a TBI-specific instrument is indicated. Several instruments for assessing health-related quality of life following TBI are available. They include the Function Independence Measure [FIM (342, 343)], the Function Independence Measure and Functional Assessment Measure [FIM + FAM (344, 345)], the Disability Rating Scale [DRS, (346)] and the Quality of Life After Brain Injury instrument [QOLIBRI (347–349)]. We recommend the QOLIBRI.

The QOLIBRI has 37 items scored on a five point scale. There are four “Satisfaction” subscales (Cognition, Self, Daily Life/Autonomy, and Social Relationships) and two “Bothered By” subscales (Emotions, Physical Problems). The subscales have high internal consistency (Cronbach alpha 0.75 to 0.89) and good test-retest reliability [interclass correlations from 0.78 to 0.85 (347, 348)]. The TOTAL QOLIBRI has an internal consistency of alpha = 0.75 and a test-retest reliability of ICC = 0.95 (347, 348). The instrument can be clinician administered or self-administered. Administration requires approximately 10 min (see Table 13).

**Table 13:** **Instruments recommended for sociological assessments**.

Domain assessed	Instrument	Reference‘
Socioeconomic status	Barratt simplified measure of social status (BSMSS)	Barratt (325)
Social support	Multidimensional scale of perceived social support	Zimet et al. (335)
Quality of life (health related)	Quality of life after brain injury (QOLIBRI)	von Steinbüchel et al. (347, 348), Truelle et al. (349)
Community integration	Community integration questionnaire	Willer et al. (351, 352)

### Community integration

The importance of community integration as an outcome measure for treatment of TBI has been considered by several authors [reviewed in (350)]. The Community Integration Questionnaire [CIQ, (351)] is self-administered, contains 15 items and has three subscales (Home Integration, Social Integration, and Productive Activities which characterizes travel, work, and training). Willer et al. (351) report good test-retest reliability and internal consistency. A subsequent study (352) established validity and showed good separation (*p* < 0.01) between TBI-positive and TBI-negative populations. The early applications of the CIQ were reported by Dijkers (353). Sander et al. (354) performed a factor analysis in a large population (*N* = 312). The three-factors identified by these calculations suggested modifications to the original questionnaire. Items on child care and shopping were deleted. The item “Who usually looks after your personal finances such as banking and paying bills?” was moved to the Home Integration subscale. The item “How often do you travel outside the house?” was moved to the Social Integration subscale.

Using the original scoring system, Zhang et al. (355) compared the CIQ against the Craig Handicap Assessment and Report Technique (356) and the Disability Rating Scale (346). They concluded that the CIQ was the most appropriate of these three instruments for characterizing post-rehabilitation community participation. Doninger et al. (357) studied the CIQ and in contrast with earlier investigators reported low reliability, poor measurement properties, and definitional problems. Reid-Arndt et al. (296) explicitly addressed the issues raised by Doninger et al. They wrote: “The validity of this measure has been suggested by several studies, including one evaluating a large sample of individuals with TBI (358) and another specifically assessing the measure’s reliability and validity (352). On the other hand, the CIQ has also been the subject of some criticism. For example, rating scale analyses have resulted in low reliabilities suggestive of poor item coherence (357) and observations have been made that the CIQ fails to account for non-TBI factors that may influence scores such as pre-injury activity levels (342) and gender (359). Despite this, results from a comparison of several outcome measures suggested that of currently available instruments, the CIQ may be the most effective measure of rehabilitation outcomes following a TBI (355).”

Dijkers (360) expanded the CIQ to produce a 47 item CIQ-2 that was used in Whiteneck et al. (361) to produce the Participation Assessment with Recombined Tools-Objective, PART-O, instrument. This instrument was used by Brenner et al. (362) in a study of health and wellness interventions for individuals with moderate to severe TBI. Insofar as we can determine, this is the only study to date to use the PART-O.

In the absence of extensive experience with the PART-O, our present recommendation is to use the Community Integration Questionnaire. It should be administered in its original form, but both the original scoring and the revised Sander et al. scoring should be reported for both the total score and the three subscales. A reassessment of this recommendation should be made after additional experience with the PART-O is published.

## Resilience

Like all psychological constructs, resilience is difficult to define in a manner that readily provides in a definition that can be assessed by a psychological instrument. As emphasized by Meichenbaum (363), resilience is not the absence of symptoms, but rather refers to a pattern of adaptation in response to stress. Castro has presented a definition that has become standard in the US military. “Resilience comprises the sum total of the psychological processes that permit individuals to maintain or return to previous levels of well-being and functioning in response to adversity” [(364); see also (365)]. Given the difficulty in defining resilience, it is not surprising that a very large number of instruments have been constructed in an effort to provide a valid and reliable assessment instrument. Several are listed in Table 14.

**Table 14:** **Instruments used in the assessment of resilience and related constructs**.

Instrument	Reference
Adolescent resilience scale	Oshio et al. (432)
Antonovsky sense of coherence scale	Antonovsky (433)
Brief resilience scale	Smith et al. (434)
California healthy kids survey resilience scale	Sun and Stewart (435)
Child and youth resilience measure	Ungar et al. (436)
Connor–Davidson resilience scale, CD-RISC	Connor and Davidson (367, 368)
Dispositional resilience scale	Bartone (437)
Ego resilience scale	Block and Kremen (438)
Ego resiliency	Klohnen (439)
Kobasa hardiness scale	Kobasa et al. (440)
Life orientation test – revised, LOT-R	Scheier et al. (441, 442)
Life satisfaction index A, LSI-A	Neugarten et al. (443)
Perceived stress scale	Cohen and Williamson (444)
Psychological resilience	Windle et al. (445)
Resilience attitudes and skills profile	Hurtes and Allen (446)
Resilience scale	Wagnild and Young (447)
Resilience scale for adults, RSA	Friborg et al. (448, 449)
Rosenberg self-esteem scale, RSES	Rosenberg (450)
Schute emotional intelligence scale	Schutte et al. (451)
Stress vulnerability scale	Connor et al. (452)
Subjective well-being scale	Ryff (453)
Youth resiliency assessing developmental strengths, YR:ADS	Donnon and Hammond (454, 455)

Windle et al. (366) reviewed 15 instruments for assessing resilience including some of those listed in the table. Based on our review, we concur with Windle et al. that there is no gold standard for assessing resilience. Windle et al. concluded that the Connor–Davidson Resilience Scale, the Resilience Scale for Adults and the Brief Resilience Scale had the best psychometric properties. Of these we recommend the Connor–Davidson Resilience Scale.

The Connor–Davidson Resilience Scale assesses 17 domains with 25 questions that are scored on a five point scale. “Not true at all” is scored as zero and “True nearly all the time” is scored as four. The maximum score is 100, and a higher score indicates greater resilience. There are also 10 question and two question versions. We recommend the 25 question version. The scores in evaluation studies [reviewed in (367)] were for the US general population 80.4(12.8), primary care patients 71.8(18.4), generalized anxiety disorder 62.4(10.7), major depression 57.1(13.3), and two PTSD populations 47.8(19.5) and 52.8(20.4).

Connor and Davidson (368) performed a factor analysis and found five factors corresponding to persistence/tenacity, self-efficacy, emotional, and cognitive control when under pressure, adaptability/ability to bounce back, and control/meaning. Subsequent studies found that the factor structure varied with setting, and therefore Connor and Davidson (367) do not recommend separate scoring of factor subscales. The scale has excellent test-retest reliability [(368), *r* = 0.87; (369), *r* = 0.70]. The 10 question version also has good test-retest properties [(370), *r* = 0.73; (371), *r* = 0.90]. An extensive literature establishing construct validity is reviewed in Connor and Davidson (367). This document also reviews studied reporting the Scale’s applications in clinical studies and in studies with military populations.

## Summary and Additional Considerations

The assessments recommended for all ERP studies are summarized in Table 15. Each of the instruments listed in Table 16 is directed to a specific disorder and is appropriate in studies where the relationship between TBI and the comorbid psychiatric disorder is of specific interest. A number of issues should be noted. Several of the recommended instruments are proprietary, and investigators should obtain appropriate access before using them. It should be remembered that the psychometric validation of an instrument is specific to the form of its presentation. If, for example, an instrument that was validated in a “paper-and-pencil” form is implemented on a computer, the previous validation studies are not, strictly speaking, applicable. Investigators will have to make a judgment as to the importance of prior validation before using an instrument. Validation concerns are particularly relevant to the acceptability of an outcome measure when studies are submitted to the Food and Drug Administration as the scientific basis for approval or clearance of an FDA regulated device or medication.

**Table 15:** **Instruments recommended for all studies of traumatic brain injury**.

Domain assessed	Instrument	Reference
Combat exposure	Combat exposure scale, or deployment risk and resiliency inventory	Lund et al. (10), Keane et al. (11), King et al. (12, 13), Vogt et al. (15)
Severity of injury at time of injury	Mayo classification for traumatic brain injury severity and/or VA/DoD classification of TBI severity	Malec et al. (31), management of concussion/mTBI working group. (17)
Current post-concussion symptoms	Rivermead post-concussion symptom questionnaire aggregate score (RPQ16) subscales: RPQ3, RPQ13, RPQ(Cognitive), RPQ(Emotional), RPQ(Somatic)	King et al. (41), Eyres et al. (42), Smith-Seemiller et al. (46), Potter et al. (47)
Current post-concussion severity	Rivermead post-concussion symptom questionnaire aggregate score	Potter et al. (47)
Assessment of general health at the time of the ERP study	Short form health survey, SF36	Ware and Sherbourne (54), McHorney et al. (55), Turner-Bowker et al. (56)
Assessment of psychiatric symptoms at the time of the ERP study	Symptom checklist-90-revised SCL-90-R	Derogatis et al. (57, 58)
Estimation of premorbid intelligence	American national adult reading test, Barona 1, Barona 2	Grober and Sliwinski (73), Barona et al. (76), Barona and Chastain (79)
Resilience	Connor–Davidson resilience scale, CD-RISC	Connor and Davidson (367, 368)

**Table 16:** **Additional instruments recommended for studies investigating traumatic brain injury and neuropsychiatric disorders**.

Domain assessed	Instrument	Reference
Depression	Beck depression inventory-II, subscales: negative self-evaluation, symptoms of depression, vegetative symptoms	Beck et al. (140), Rowland et al. (152)
Post-traumatic stress disorder	First screen: PTSD checklist (PCL), if positive: clinician administered PTSD scale	Weathers et al. (187), Bliese et al. (411), Blake et al. (179, 182, 183)
Anxiety	Beck anxiety inventory	Beck et al. (153)
Psychotic disorders	Scale for the assessment of negative symptoms, scale for the assessment of positive symptoms	Andreasen (218), Andreasen (217)
Sleep disorders	Pittsburgh sleep quality inventory, global score and seven component scores	Buysse et al. (228)
Suicidal ideation	Beck hopelessness scale	Beck et al. (456), Beck and Steer (457)
Alcohol abuse	Alcohol use disorders identification test, AUDIT	Babor et al. (269)
Substance abuse	Drug abuse screening test, DAST	Skinner (286)

Some of the recommended instruments assess suicidal ideation, uncontrolled outbursts of temper, and thoughts of injuring others. Investigators have a responsibility to respond if a participant discloses thoughts of injury to self or others. The form of this response will vary according to the qualifications of the investigators and the location of the study. For example, support resources such as emergency psychiatric consultations that are available in a teaching hospital will not be available in academic departments and schools. The specific legal requirements placed on investigators will vary. Typically, a response plan should be in place and approved by the investigators’ Institutional Review Board (Human Subjects Protection Committee) before initiating the investigation.

## Conflict of Interest Statement

The authors declare that the research was conducted in the absence of any commercial or financial relationships that could be construed as a potential conflict of interest.
